# Theatres of surgery: The cultural pre-history of the face transplant

**DOI:** 10.12688/wellcomeopenres.14558.1

**Published:** 2018-05-08

**Authors:** Suzannah Biernoff

**Affiliations:** 1 Department of History of Art, Birkbeck, University of London, London, WC1H 0PD, UK

**Keywords:** Face transplantation, organ transplantation, plastic surgery, disfigurement, Frankenstein, Georges Franju, Face/Off

## Abstract

The first facial transplant, using a donor’s nose, chin and mouth, was performed on Isabelle Dinoire in France in 2005, but the idea of removing or replacing the face – either with a mask, or with a living face – has been around for much longer. This article explores the cultural pre-history of face transplantation: its speculative existence in legend, literature and film before it became a medical possibility at the beginning of the twenty-first century. One of the questions posed here is: how (and for what purpose) do medical ‘firsts’ like Dinoire’s surgery acquire a history? The article begins by considering the uses of the past by transplant surgeons themselves, and by those who are concerned about the ethical or psychological implications of organ and face transplantation. Having considered these different investments in the past – one emphasising medical progress, the other highlighting enduring anxieties about medical experimentation – we turn to the first cinematic portrayal of face transplantation, in Georges Franju’s horror classic *Les Yeux sans Visage* ( *Eyes Without a Face*, 1959). An exploration of Franju’s sources suggests a more complicated relationship between medical innovations and their cultural contexts and highlights the changing significance of the face as a site of medical and aesthetic intervention.

## Introduction

This article begins with two versions of the history of the face transplant. Transplant surgeons often see themselves as inheriting a tradition of surgical innovation stretching back through Renaissance Italy to ancient India, classical Greece and classical era China. Key figures in this genealogy include the sixteenth-century surgeon Gaspare Tagliacozzi (1545-99), the early Christian saints Cosmas and Damian, often named as the founding fathers of transplant surgery, and the legendary Chinese physician Pien Ch’iao (Bian Qiao) who lived in the 5 ^th^ century BCE. In a 2007 interview with Simon Hattenstone for the *Guardian*, the British plastic surgeon Peter Butler claimed both an ancient lineage and a moral imperative for face transplantation. Explaining that ‘plastic surgery goes back thousands of years,’ he insisted that there was nothing ‘revolutionary’ about repairing the face using tissue from another person. The procedure presents logistical, medical and psychological challenges, but for the severely disfigured, transplantation is simply a better way of doing what surgeons have been trying, with less success, to do for millennia ^1^.

In the course of their conversation, Butler shows Hattenstone a picture illustrating a forehead graft technique and surgical instruments in use in ancient India. Other images, unseen but carefully described, play an equally significant role in the story. The interview itself takes the form of a ‘guided tour of the facially disfigured’, and although we don’t see Butler’s case photographs, we have his direct commentary (‘Here we've got a guy who had his nose taken off. He came across someone wielding a samurai sword – not a good idea on a Saturday night’) and Hattenstone’s reactions (‘There's just a hole where a nose should be – awful’). A framed photograph of Joseph Merrick, the ‘Elephant Man’, hangs on the wall in the surgeon’s office on the ninth floor of the Royal Free Hospital in Hampstead. Medical illustrations, clinical photographs and historical relics perform different rhetorical functions, of course: images from ancient medical texts demonstrate that the idea of restoring or replacing the face is not new; Butler’s graphic case histories show the limitations of conventional plastic surgery and personalise the patients for whom transplantation remains an unfulfilled hope. The portrait of Merrick posing ‘like a model’ in his newly painted attic flat is harder to read. It bears the simple dedication to Merrick’s physician Frederick Treves: ‘Dear Freddie, a thousand thanks for the whitewash.’

This story of human tragedy, scientific innovation and surgical salvation is exhibit one. Exhibit two appears more obliquely in Hattenstone’s *Guardian* article, when he asks Butler about the obstacles that have delayed Britain’s entry into the face transplant race. By 2007 there had been three successful partial face transplants, two in France and one in China. Butler and his team at the Royal Free had been planning the world’s first full-face transplant for 15 years. ‘Not surprisingly,’ reflects Hattenstone ‘he has met with resistance. After all, this is the stuff of Gothic horror, grave robbers and Victorian freaks shows’ ^2^.

Between 1991 and 1993 the Park Ridge Center for the Study of Health, Faith and Ethics in Chicago funded an interdisciplinary project that culminated in a volume of essays entitled *Organ Transplantation: Meanings and Realities* (1996). In the personal statement that prefaces his contribution, the pioneer of disability studies Leslie Fiedler recalls that when he was invited to give a literary perspective on organ transplantation he instinctively reached for the classics of nineteenth-century horror: ‘almost without thinking, I took down from my shelf the well-thumbed copies of two of the most popular of all popular books, *Frankenstein* and *Dracula*.’ ^3^ The particular nightmares conjured by these stories (Fiedler’s other examples are *The Island of Doctor Moreau* and *Dr Jekyll and Mr Hyde*) are symptomatic, he suggests, of modernity’s unease with the pact it has made with medical science ^4^.

Fiedler’s reflections don’t extend to face transplantation – it would be several years before the international news media started reporting on the face transplant race – but his instinctive recourse to Mary Shelley’s *Frankenstein* is not uncommon in academic and journalistic discussions of face transplantation. In her autobiography *Transplanting a Face: Notes on a Life in Medicine* (2008), Maria Siemionow expresses concern about ‘overly dramatized’ accounts of face transplant surgery unleashing a ‘Frankenstein syndrome’ ^5^. In 2004 French surgeons Francois Petit, Antoine Paraskevas and Laurent Lantieri warned that the association with ‘the Frankenstein story’ could undermine public acceptance of face transplantation and lead to a shortage of potential donors. ‘Care should be taken to not frighten or repulse the population’ they urged in *The American Journal of Bioethics*’ ^6^. Falklands War veteran Simon Weston offered a more phlegmatic view in the 2006 BBC *Horizon* documentary, *The World’s First Face Transplant*. ‘The healing of people has always been about making dangerous, so-called dodgy, scary, frightening, almost Frankenstein decisions,’ he says. ‘That’s the way medicine’s had to proceed for centuries’ ^7^.

The first part of this article considers the implications of these different approaches to the past; one serving to legitimise experimental surgery, the other foregrounding the ethical questions and cultural anxieties provoked by the transfer of body parts from one person to another. Much of this commentary pre-dates Dinoire’s operation and occurs in the academic literature on organ transplantation. Since 2004, when *The American Journal of Bioethics* published its special issue on face transplantation, similar concerns have started to surface in discussions of face transplantation and in the extensive press coverage of Dinoire and other recipients. What most of these accounts lack is an understanding of myth and legend – from Cosmas and Damian’s ‘miracle of the black leg’ to *Frankenstein* – as narratives that take on different forms and acquire new meanings through time.

Indeed, Fiedler insists that ‘mythic works’ such as Shelley’s *Frankenstein* ‘exist out of time, in the eternal now of the collective unconscious’ ^8^. The evidence assembled here, however, suggests a more complex, evolving relationship between medical ‘progress’ and cultural production. Rather than seeking to probe the collective unconscious or to reveal universal themes, the second half of this article plots a cultural history of the face transplant through a film that acknowledges but radically refigures the familiar canon of Gothic literature: Georges Franju’s *Les Yeux sans Visage* ( *Eyes Without a Face*, 1959). Franju both exploits and extends the symbolic and aesthetic territory of the modern ‘surgical imaginary’: a term used by the historian Susan Lederer to indicate that ‘the body and its parts – organs, tissues, cells, and fluids – possess not just medical and surgical significance, but complex political and cultural meanings as well’ ^9^. In her book *Flesh and Blood: Organ Transplantation and Blood Transfusion in Twentieth-Century America* (2008), Lederer suggests that one of the functions of modern films and novels has been to establish ‘a trajectory of possibility about remaking the human body.’ Horror movies, early science fiction and pulps like *Detective Weekly* and *Horror Stories* ‘provided representations of surgical science; the buying, selling, and stealing of blood and organs; and the social, cultural, and political implications of these techniques’ ^10^.

Lederer gives more weight to these varied cultural representations than most historians of medicine, but the examples she mentions – from *The Island of Doctor Moreau* (1896) to *The Hero* (1923), *Dr. Renault’s Secret* (1942), *The Ape Man* (1943), and *Coma* (1978) – are discussed only briefly. This article borrows her concept of the surgical imaginary, but returns to the original understanding of what ‘imagining’ entails (from the Latin *imaginari*, to picture to oneself). How was face transplantation visualized in the decades before it became a medically and ethically viable procedure? Does the ‘surgical imaginary’ have iconographic or aesthetic attributes? To simply précis a film’s plot is to miss the significance of visual representation and ways of seeing in the history of medicine. If the task is to understand how the surgical imaginary works – how it gets under your skin – one needs to know how facial transplantation it is pictured, and to what effect, as well as what its cultural and political meanings might be.

## Making history: ‘the new divine healers’

In a review of the 17 face transplants performed between 2005 and 2012, Lantieri (who had been involved in 7 of the procedures) pronounced a ‘paradigm change in facial reconstruction’ ^11^. His article begins by looking back through history at the ‘slow natural progression’ of reconstructive surgery punctuated by ‘sudden profound change’:

The forehead and facial flaps first described in Sushruta Samhita in 600 BCE were passed down for generations until a compendium of facial reconstructive procedures was compiled by Vagbhat in the fourth century CE. These ancient Indian surgical procedures were carried into Greece and Arabia by Buddhist missionaries during the Middle Ages. At the Persian hospital of Gondi-Sapor (6th-10th century CE), Hindu, Greek, and Arab surgical principles were unified, and a new school of thought moved west. By the middle of the Renaissance, Gaspare Tagliacozzi had promulgated the Eastern techniques into Europe. From there, the westward expansion of surgical knowledge passed north through France, Germany, and England, and for a time, the advancements in surgery seem limited only by the imagination ^12^.

Many articles and books by reconstructive surgeons begin much the same way, with founding fathers, technical or conceptual breakthroughs, and lines of descent. Transplant surgery, too, has its list of greats and firsts: Siemionow begins her history lesson in China some 2,500 years ago, when the physician Pien Ch’iao is said to have ‘exchanged the heart of a man with a strong spirit but weak will with the heart of a man who had a weak spirit but strong will.’ ‘This is a myth, of course,’ she adds, ‘but the story has value. It shows that 2,500 years before the first heart transplant was conducted, men were thinking that it was possible to use one individual’s tissues and organs to restore another’s’ ^13^.

One of the interesting features of transplant historiography is that it so often recruits mythical figures and events. In *Encyclopedia Britannica*, the idea of transplantation goes all the way back to the Genesis story of the ‘creation of Eve from one of Adam’s ribs’ ^14^. The chapter on organ transplantation in R. H. Mead’s *An Introduction to the History of General Surgery* (1968) lists the Chimera, the Minotaur and ‘the wings of Daedalus and Icarus’ as evidence that ‘the possibility of successful organ replacement has challenged men’s minds through the ages’ ^15^. The ancient Greek figure of the Chimera reappears on the cover of Najarian and Simmons’ *Transplantation* (1972). This ‘fabulous monster – part lion, part goat, and part serpent – has come to represent a successful transplant of genetically foreign tissue’ explain the authors in the Preface ^16^. Siemionow’s own choice of cover image is equally suggestive of a tradition linking modern biomedicine to ancient myth: a Mycenaean death mask formed out of a single hammered sheet of metal foil, a malleable golden skin.

In his article ‘How gods and saints became transplant surgeons,’ the historian Tomas Schlich asks why surgeons ‘bother with history’ at all ^17^. He notes that the historical accounts given by transplant surgeons tend to rely on the same methods of persuasion – the same style, rhetoric and structure – as scientific papers ^18^. One of the key rhetorical tools in this literature is genealogy. Past successes and failures are ‘measured against present day standards,’ and the lesson of history is always that science progresses ^19^. By aligning current goals with past practices and beliefs, the present acquires a kind of inevitability: ‘Transplantation appears to be something that had already existed before modern surgery invented it’ ^20^.

Bruce Conolly and Mario Benanzio’s opening reflections in *Hand Transplantation* – a major reference volume published in 2007 – focus on a legend mentioned in almost every account of the history of transplantation: the miracle of the black leg ^21^. The story involves two brothers, Cosmas and Damian, who lived in Asia Minor during the reign of Emperor Diocletian (245–316). Their fame as physicians – and refusal to take payment for healing the sick – came to the attention of the Roman proconsul, who demanded that they make a sacrifice to the gods. When the brothers refused, the proconsul had them thrown into a fire, but they did not burn. Attempts to crucify them, stone them to death and slay them with arrows also failed: the stones and arrows were flung back at their tormentors. Finally decapitated, Cosmas and Damian became Christian martyrs. In the centuries after their deaths reports of their miraculous works proliferated: the canonical listings include cures for ‘plague, scabs, scurvy, glandular problems, kidney stones, abdominal swelling, and bedwetting’ ^22^.

The miracle recounted by Conolly and Benanzio makes its first appearance around the twelfth century ^23^. In Jacopus de Voragine’s *Golden Legend*, compiled around 1260, the scene is a Roman church built by Pope Felix in honour of the martyred brothers. A man suffering from cancer (or gangrene) of the leg has taken refuge in the church, devoting himself to the saints’ service. One night as he sleeps they appear with their salves and instruments:

One of them says to the other: “Where can we get flesh to fill in where we cut away the rotted leg?” The other said: “Just today an Ethiopian was buried in the cemetery of Saint Peter in Chains. Go and take his leg, and we’ll put it in place of the bad one.”

On waking, the man finds himself cured, but with a black leg, and tells everyone he meets of the extraordinary dream he has had and how he has been healed. When the Ethiopian’s tomb is opened, the diseased leg is discovered attached to the body of the dead man ^24^.

In the legend, Cosmas and Damian perform the miracle as saints, after their martyrdom, but what happens in the various retellings of the story, and its re-imagining in art, is that the miraculous and the real start to mingle. *From Laughing Gas to Face Transplants* – an illustrated volume aimed at children – includes Fra Angelico’s fifteenth-century depiction of the miracle on the first double-page spread. ‘That’s Amazing!’ exclaims the text box: ‘Limb transplantation may not be as new as you think’ ^25^. The art historian Leonard Barkan notes that many Renaissance artists rendered the surgery ‘with grisly realism, which is quite in keeping with the canons of holy image making, especially outside Italy’ ^26^. In a late sixteenth-century painting by Ambrosius Francken the Elder, reproduced in Conolly and Benanzio’s chapter, the severed leg, bloody stump and surgical accoutrements are arranged for inspection much as the wounds of Christ and instruments of torture are presented to the viewer in paintings of the Passion. Some accounts have the saints covering their noses because of the stink of the diseased leg.

Barkan’s point is that the ‘medical precision’ of such paintings needs to be understood in its historical context ^27^. The corporeality of the miracle – its convincing realism – ‘becomes a subcategory (like many other things) of what it means to have a human Christ’ ^28^. There is a larger cautionary lesson to be learned here. The flesh-and-blood realism of the paintings – indeed any work of art – is both symbolic and instrumental: in the case of medieval saints and their miracles, it brings the divine into the human sphere. Transplant surgeons are typically unconcerned with such theological nuances. For Conolly and Benanzio, the legend is simply ‘proof that the idea has existed for a long time’. What was once only an idea – a ‘dream of humankind’ – is now a reality. Today’s transplant surgeons, they conclude, are the ‘new divine healers’ ^29^.

## Gifts, thefts and sympathetic noses

All historical narratives are selective, but the ones discussed briefly here present a singular, implicitly universal idea of transplantation that they trace back to the dawn of human civilization. The function of these accounts, as Schlich argues, is to naturalise and legitimise transplant medicine. There are, however, other legends about the giving and taking of body parts that don’t feature in accounts of transplant surgery, and that unsettle the conviction that transplantation is a shared ‘dream of humankind’. The best-known examples are associated with Gothic literature and its recurring scenes of iatrophobia (the fear of doctors or medical procedures). But there are older legends too, with diverse geographical origins, such as the Inuit story of the mother who steals her daughter’s face in order to deceive her son-in-law ^30^. Annie Dillard’s retelling of the story conveys its rootedness in a specific material culture and oral tradition:

A young man in a strange land falls in love with a young woman and takes her to wife in her mother’s tent. By day the women chew skins and boil meat while the young man hunts. But the old crone is jealous; she wants the boy. Calling her daughter to her one day, she offers to braid her hair; the girl sits pleased, proud, and soon is strangled by her own hair. One thing Eskimos know is skinning. The mother takes her curved hand knife shaped like a dancing skirt, skins her daughter’s beautiful face, and presses that empty flap smooth on her own skull. When the boy returns that night he lies with her, in the tent on top of the world. But he is wet from hunting; the skin mask shrinks and slides, uncovering the shrivelled face of the old mother, and the boy flees in horror, forever ^31^.

A different set of concerns animates the Buddhist version of the story of King Shivi from the *Shiva Jataka*. In an exemplary act of selflessness, the king resolves to give a part of his body to anyone who asks for it. Indra, the king of the gods, decides to put Shivi to the test. Assuming the form of an old, blind Brahmin, he asks for one of the king’s eyes. Immediately, Shivi calls for his surgeon and instructs him to remove both eyes. As in the Inuit legend, the details here are precise and salutary: ‘The king’s garments were stained with blood, but he endured the pain and simply said, “My friend, be quick.” The surgeon grasped the eyeball with his left hand, took a knife in his right hand, and severed the tendon and laid the eye in Shivi’s hand’ ^32^. After the king has fulfilled his promise, Indra reveals his true identity and restores Shivi’s sight. As Wendy Doniger points out, the story is in keeping with the Buddhist denigration of the physical body, but its realism also emphasises the king’s courage and generosity ^33^.

The meanings of transplantation are culturally specific, but even within a given culture there are competing ideas about the limits, properties and proprieties of human embodiment. Tagliacozzi’s unexpectedly comic legacy is a case in point. The renowned maker of noses and professor of surgery at the University of Bologna declared in his 1597 treatise that a surgeon’s task is to ‘restore, repair, and make whole those parts of the face which nature has given but which fortune has taken away, not so much that they might delight the eye but that they may buoy up the spirits and help the mind of the afflicted’ ^34^. He is credited, in most histories of plastic surgery, with introducing the pedicle graft into Europe, a technique that uses a flap of skin from the patient’s own arm to create a new nose. A statue of him holding a delicately crafted nose in his hand, carved by Silvestro Giannotti in 1734, stood in the Anatomical Theatre in Bologna until it was bombed in 1944 ^35^.

Although the procedure for which Tagliacozzi was famous did not rely on donor tissue, by the seventeenth-century there were several accounts of his surgical exploits with noses being fashioned from other people’s arms, noses or – in Samuel Butler’s comic poem *Hudibras* (1662-63) – their backsides:

So learned *Taliacotius* from
The brawny part of Porter’s Bum,
Cut supplemental Noses ^36^

Apocryphal reports of slaves’ noses being used to restore their masters’ faces were already in circulation in fifteenth-century Italy, though Tagliacozzi himself pointed out the impracticality of binding two people together for long enough for a graft to adhere ^37^. By the early eighteenth century, the missing nose was widely recognised, and stigmatised, as a symptom of the pox. As Emily Cock discusses in a fascinating essay on rhinoplasty in the long eighteenth century, the trope of the absent and reconstructed/transplanted nose provided satirists with ‘a short-hand for lewdness’. One story, published in the *Tatler* in 1710 and recounted by Cock, concerns three Spanish noblemen with noses ‘all made out of the same Piece of Brawn’ (the same porter’s bum). One day they feel their noses ‘shoot and swell extremely’ and, investigating the cause of the malady, discover that the porter had received a painful beating on his bottom – an injury transmitted sympathetically to their noses ^38^.

Surgical innovations enter popular culture in many different ways and forms: as medical miracles, cautionary tales, horror stories and as grist to the satirist’s mill. They make us marvel, shudder and laugh, sometimes at the same time. One of the unspoken assumptions in the few examples included here is that the donor or donor part is always ‘other’ – either racially other, as in the miracle of the black leg, or other in terms of corporeal or social hierarchies (with noses crafted from lower-class arses or transferred from slaves to their masters). Modern transplant stories, both fictional and factual, often turn instead on an intimacy or identification between donor and recipient: a disturbing lack of otherness that unsettles the biomedical model of replaceable ‘spare parts’ ^39^.

## Iatrophobia

‘Why, despite our avowals to the contrary, do so many of us not give the much touted “gift of life?”’ asks Fiedler in his essay ‘Why Organ Transplant Programs Do Not Succeed’. As he re-reads the classics of Gothic literature – Mary Shelley’s *Frankenstein* (1818), Bram Stoker’s *Dracula* (1897), Robert Louis Stevenson’s *Strange Case of Dr Jekyll and Mr Hyde* (1886) and H. G. Wells’ *The Island of Doctor Moreau* (1896) – an answer begins to takes shape. Victor Frankenstein’s experiments into galvanism are conducted in a university laboratory rather than an operating theatre, but in countless re-imaginings of the story (one filmography lists 57 adaptations) ^40^ Dr Frankenstein becomes ‘the prototype of the modern white-jacketed surgeon transplanting hearts and livers’ ^41^. In *Dracula*, the ‘irruption of vampirism into the modern world’ gives allegorical form to anxieties surrounding blood transfusion (a risky procedure until the early 1900s, when blood typing and cross-matching of donor and recipient were introduced) ^42^. Dr Jekyll’s self-administered psychochemical experiments release ‘all the dark impulses we normally repress for the sake of civility’ ^43^, and on Dr Moreau’s island vivisection is practiced as a form of accelerated surgical ‘evolution’, as animals are cruelly disassembled and reassembled into quasi-human form.

The horrors these novels confront us with, Fiedler argues, are born of modern science and technology, particularly medical technology ^44^. Their monsters and mad and misguided scientists have become part of a ‘collective consciousness’ – shaping the stories we tell and the beliefs we hold – but they also inhabit a collective unconscious capable of ‘triggering’ psychological responses that, in turn, influence our behaviour. Each generation re-makes these tales, adding and subtracting elements, but, he insists, their ‘mythological core’ remains ^45^. Robert Silverberg’s *Caught in the Organ Draft* (1972) and Larry Niven’s *The Jigsaw Man* (1967) and *The Patchwork Girl* (1980) are cited as more recent examples of the genre, but for Fiedler the organ harvesting scenarios played out in contemporary science fiction ‘seem updated versions of familiar nineteenth-century tales about “resurrection men”, the bodysnatchers who provided corpses for dissection in anatomy classes of medical schools and hospitals’ ^46^. Dr Frankenstein is still the ‘archetypal doctor’ who dares to ‘usurp the prerogatives of a superhuman creator’. The unnatural progeny of modern science are still monstrous and tragic.

The psychiatrist Stuart Youngner, another participant in the Park Ridge Center project, warns that we ignore the ‘dark side’ of organ procurement at our peril. ‘There seems always to be lurking in the public consciousness a suspiciousness and paranoia that defies the rationality of medical science,’ he writes, citing *Frankenstein, Bride of Frankenstein* (1935) and Michael Crichton’s *Coma* (1978) as evidence of this collective unease. More recently, clinical psychologists Carla Bluhm and Nathan Clendenin have argued that the Frankenstein myth is ‘an inevitable backdrop for the staging of the world’s first face transplant procedure’:

Regardless of the murkier issues that it stirs up, it is the tale of a doctor animating the dead through technology. … Being a profound meditation on the nature of monstrosity, the Frankenstein myth even offers the opportunity (as is especially apparent in the early [1931] film version of *Frankenstein* starring Boris Karloff) to question what makes a monster: the nature of the beast or the fears that reside within us? ^47^

Fiedler’s characterisation of the ‘mythological core’ of these canonical works of nineteenth-century fiction is not unusual, then, but is it ultimately any different from the claim that the dream of transplantation has always existed? Both of these approaches – one pro-transplant surgery, the other averse to it – take historical sources and use them to make an ahistorical argument. Lederer, who considers several examples of the cultural aversion thesis, points out that accounts of skin grafting and blood transfusion from the early 1900s present a far more complex picture, with many Americans seemingly quite willing to ‘go under the knife’’ ^48^. Bioethicists sometimes speak of the ‘yuck factor’, a term coined by Arthur Caplan in the 1980s ^49^, but Lederer insists that ‘the history of medicine, and of organ transplantation in particular, defies assumptions that the repellant is a historically stable category’ ^50^.

These sources highlight the problem with using cultural artefacts – folklore, novels, paintings, films – as straightforward evidence of social attitudes. The following discussion of *Les Yeux sans Visage* treads a more cautious path. Films, like any art form, can be idiosyncratic, controversial and culturally marginal; they can be dismissed by the critics, but commercially successful, or acquire a cult following decades after their original release. Art divides opinion as much as it reflects or creates it. And the interpretations one has access to – usually through interviews with directors and actors, or published film reviews – are not necessarily reliable or representative. In what sense, then, is a film, a novel or a photograph a mirror of its time? *Les Yeux sans Visage* certainly reflects Franju’s own fascination with surgery as a primal scene – a wellspring of both horror and beauty – but it also exploits historically specific anxieties about medical experimentation, while paradoxically paying homage to scientific (including surgical) films. ‘Did Franju himself really know what he was up to?’ pondered Robert Vas in his 1960 review for *Sight & Sound* ^51^. Possibly not, but the film’s ability to baffle, enrage, sicken, excite and disturb audiences makes it well suited to the kind of ‘unfettered cultural history’ that Roger Luckhurst has called for in science fiction studies: ‘a science fiction in the expanded field, traced not by pre-existent categories, but following how the actors construct the pathways through the messy culture they constantly sift and sort’ ^52^.

## Eyes Without a Face

On the surface, *Les Yeux sans Visage* looks like a classic example of iatrophobia: a chilling reflection on the limits of medical science that coincides with the first successful organ transplants of the 1950s and 60s ^53^. Plastic surgeons and facial disfigurement had already been established as elements of horror cinema in films like *The Raven* (1935), *The Stolen Face* (1951), *La Bruja* ( *The Witch*, 1954), and *Circus of Horrors* (1959) ^54^. There had been cinematic hand transplants in *Mad Love* (1935) and other versions of Maurice Renard’s novel *Les Mains d’Orlac*; glandular transplants and transfusions ( *The Man in Half Moon Street*, 1944; *She Demons*, 1958) and brain transplants ( *The Monster and the Girl*, 1941; *The Ape Man*, 1944; *The Revenge of Frankenstein*, 1958). Franju’s film brings these winning ingredients together in a film that has been credited (along with Hitchcock’s *Psycho*, released the same year) with ‘co-paternity of the splatter genre’ ^55^.

Pierre Brasseur plays Dr Génessier, an eminent plastic surgeon whose daughter Christiane (Edith Scob) has been horrifically disfigured in a car accident. With the help of his assistant Louise (Alida Vallie), the professor attempts to restore his daughter’s appearance using the faces of kidnapped young women. During the film’s world première at the 1959 Edinburgh Film Festival, several members of the audience reportedly fainted watching the face removal scene ^56^. Unimpressed by Franju’s avant-garde credentials, British critics were generally appalled. In her review for *The Sunday Times* in September 1959, Dilys Powell called the film ‘deliberately revolting’ ^57^.

During the opening credits we see Louise driving at night along a deserted road, glancing anxiously in her rear view mirror at something, or someone, on the back seat and stopping to drag her passenger — clearly now the body of a girl — into a river. When Génessier turns up at the morgue and identifies the faceless corpse as his daughter we realise that the professor and his assistant are co-conspirators. The detectives assigned to the case believe that Christiane went missing from her father’s private clinic, but the suicide hypothesis leaves disturbing questions unanswered. The corpse’s ‘vast open wound in place of a face’ has edges ‘so clean that it might have been done by a scalpel.’ And despite this gruesome injury — which we mercifully don’t see — the girl’s eyes are undamaged.

**Figure 1.**
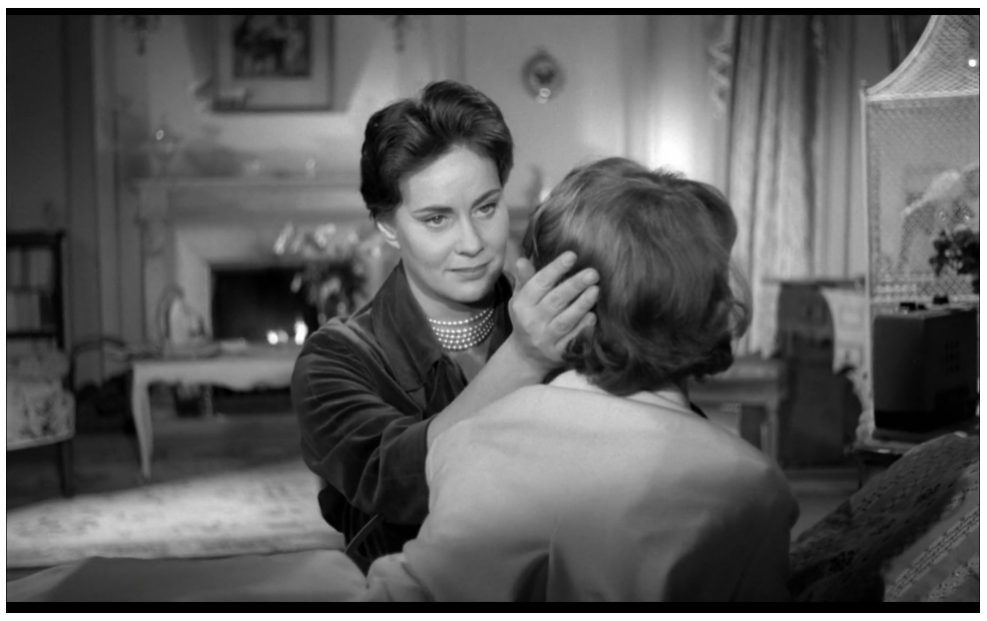
Georges Franju, *Les Yeux sans Visage*, 1959. Screenshot.

The real Christiane, when we encounter her for the first time, is lying on a *chaise longue,* her face buried in a pillow. Birds flutter in an ornate cage, music is playing on a wireless, a fire burns in the hearth. She has discovered the announcement of own funeral. Génessier strokes his daughter’s hair and reprimands her gently for taking off her mask. ‘I am not allowed mirrors, but I can see my reflection in the window,’ she whispers. ‘If the windows are open, there are other shiny surfaces … the blade of a knife, polished wood. My face frightens me, my mask frightens me even more.’ With her back to the camera, she slowly sits up and turns to face Louise, who tenderly lifts the white mask and fixes it in place ( Figure 1). 

## Homo biologicus

*Eyes Without A Face* is often described as a modern reworking of *Frankenstein*, but the character of Dr Génessier has historical as well as fictional predecessors. As Jon Turney observes in *Frankenstein’s Footsteps*, French scientists have often been controversial figures. His list of real-life Victor Frankensteins includes the anatomist Marie-François-Xavier Bichat (1771–1802); Claude Bernard (1813–1878), who was a famous defender of vivisection; and the Nobel Prize winning physiologist and eugenicist Alexis Carrel (1873–1944) ^58^. Carrel, who was no stranger to self-publicity, re-enacted Cosmas and Damian’s miracle at New York’s Rockefeller Institute of Medical Research, grafting a white leg onto a black dog and a black leg onto a white one ^59^. Writing about Carrel’s transplant experiments in *Collier’s* magazine in 1912, science journalist Carl Snyder made a plea for a ‘cold storage facility’ capable of storing organs and limbs for transplanting. ‘Depending upon the degree of mutilation’, he mused – ‘whether the bodies are blown to pieces, or chewed up, or merely punctured by a bullet, or killed electrically,’ there must be ‘at a modest calculation … at least 50,000 good arms, as many legs, and perhaps a slightly less number of lungs, livers, hearts and other organs’ ^60^.

There is a more immediate resemblance between Génessier and the celebrated French biologist Jean Rostand (1894-1977), whose book *Peut-on Modifier L’Homme?* ( *Can Man Be Modified?)* was published in France in 1956 and in English translation three years later ^61^. Hailing the birth of a new ‘Scientific Age’, Rostand anticipates a future in which human potential is dictated not by nature but by the ‘magic wand’ of science: ‘Here and now,’ he proclaims, man ‘is changing into a new and paradoxical animal. … *Homo sapiens* is in the process of becoming *Homo biologicus* ^62^. Part of the book deals with organ transplantation and Rostand responds to the ethical concerns raised by the jurist Aurel David, whose *Structure de la Personne Humaine* had been published the previous year. David had condemned the use of cadavers for ‘spare parts’ as a ‘moral revolution’ with profound legal implications. Is an individual who receives someone else’s organ ‘still strictly himself?’ David had asked, ‘and could he not say, like the poet: *Je est un autre*? And what view are tradition and the law to take of this cavalier way of treating living fragments of the person as inanimate objects, as common things?’ The very concept of the person, David warned, was on the point of ‘losing its naturalness’ ^63^.

Far from being horrified by this prospect, Rostand embraced it:

Quite soon, perhaps, people will buy genius or sanctity at the chemist’s, just as women now buy the straightness of their nose or the depth of their gaze at the beauty parlour. … “ *Denatured Men*” … what a fine book for a future Vercors to write! ^64^

The film critic Raymond Durgnat noted in his 1967 book on Franju that the facial graft performed by Génessier is ‘currently on the border of science fact and science fiction’. Is it possible, he wondered, ‘that we might one day have facebanks as well as bloodbanks and eyebanks, or even standardized faces which the body and mind would slowly remould and reinterpret?’ ^65^ Driven by guilt and distorted by self-belief, Génessier has none of Rostand’s charisma, but at the start of the film, when he is describing a future without ageing or ugliness to a roomful of well-heeled Parisians, we glimpse the seductive possibility of conquering nature. One woman in a fur coat and pearl earrings has brought her poodle. A Jesuit priest sits in the front row. ‘Man’s greatest new hope,’ Génessier begins, ‘is the recapture of physical youth. This hope is afforded by the heterograft … that is to say the transplanting of living tissues or organs from one human being to another’ ^66^. ‘Thrilling, wasn’t it?’ enthuses one of the guests after the lecture. ‘What a wonderful future you showed us professor!’

Animal experimentation is rarely mentioned in the context of face transplantation, but Durgnat remarks on the ‘eerie echo of Franju themes’ in an anti-vivisectionist photograph he had seen, of a dog with a second head grafted onto its neck ^67^. Génessier’s procurement of stray dogs is one of the most disturbing aspects of the film and we hear the animals barking and whimpering dementedly long before we see them. When Christiane’s graft fails, Génessier returns to his ‘laboratory’, a windowless cellar lined with individual cages. It is, as Joan Hawkins and other critics have noted, the suburban equivalent of Dr Moreau’s ‘House of Pain’ ^68^. As he inspects one of his patchwork creations, Génessier wonders why the grafts take on the animals, but not on his daughter. In a sense, they are all his creatures. ‘He’ll experiment on me as if I were one of his dogs. A human guinea-pig,’ despairs Christiane. Fearing that the operations will never end, she begs Louise to give her the lethal injection used on the animals when things go wrong. Louise – Génessier’s most faithful and devoted companion – refuses. Although the details of her own surgical transformation are never revealed, she hides the scar with a pearl necklace wound around her neck ‘like a dog collar’.

**Figure 2.**
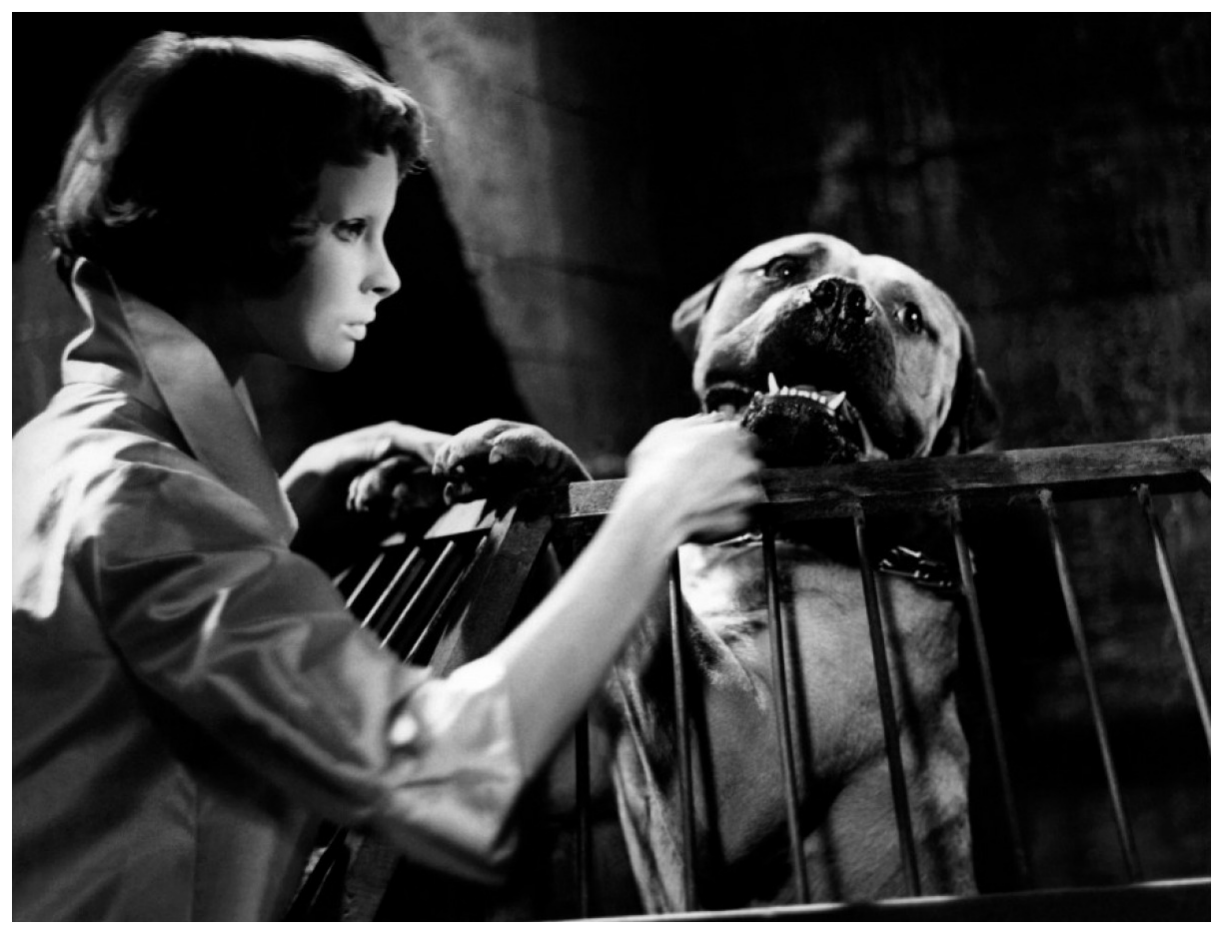
Georges Franju, *Les Yeux sans Visage*, 1959. Screenshot.

Adam Lowenstein suggests that for contemporary French audiences, the sight of the dogs – which include an enormous German Shepherd – and the sound of their frenzied barking would have triggered memories of Nazi occupied France ^69^. But in the context of the film, the dogs are not tools of intimidation or oppression: they are victims of medical experimentation ^70^. Christiane’s identification with and affection towards them further complicates Lowenstein’s reading, which posits historical trauma as the collective ‘wound’ allegorically embodied in horror cinema ^71^. When Christiane visits her father’s laboratory alone, the dogs stop barking and allow her to caress their faces ( Figure 2). Kate Ince points out that the use of ‘swift but compassionate’ close-ups as the camera moves from cage to cage has the effect of ‘facialising and humanising’ the animals ^72^. At the end of the film Christiane opens the cages and the liberated dogs turn on their tormentor with eye-watering savagery as he attempts to flee into the woods. Génessier is left mutilated beyond recognition in an act of collective vengeance. Christiane’s final symbolic gesture is to release the white doves her father keeps in a cage. One settles on her hand, the others flutter around her, their feathers falling like snow. 

## Scientific realism and surgical spectacle

Despite its obvious parallels with *Frankenstein* and *Doctor Moreau*, *Eyes Without a Face* is not really a cautionary tale of scientific hubris and its grotesque consequences. Ultimately, Franju seems more interested in the aesthetic possibilities of his disturbing subject matter than in any moral to be drawn from it. One of his favourite anecdotes involved a projectionist he had worked with at the Museé de l‘Homme in Paris and bumped into many years later. ‘M. Franju!’ exclaimed the projectionist, ‘we haven’t seen each other for 19 years. Do you remember that surgical film which had twenty people flat out on the floor?’ ‘That was an authentic horror film,’ Franju replied, ‘I’ve never seen anything so drastic. It was an atrocious film, but a beautiful and poetic one, because it was so realistic’ ^73^.

Made in 1940, the film (now lost) was Dr Thierry de Martel’s *Trépanation pour crise d’epilepsie Bravais-Jacksonnienne* (Trepanation for a Bravais-Jacksonian epileptic seizure) ^74^. Edith Scob and Bernard Queysanne recalled Franju talking about it on many occasions. He was, Scob says, ‘passionately interested in surgery and medicine’ and spoke ‘beautifully’ about the film ^75^. The strange juxtaposition of irreconcilable images — the patient’s open skull, his beatific smile — was clearly part of the film’s appeal, as was the element of suspense. Unable at first to see the patient’s face, and unaware that local anaesthetic meant that he felt no pain, the spectator imagined the most awful suffering.

**Figure 3.**
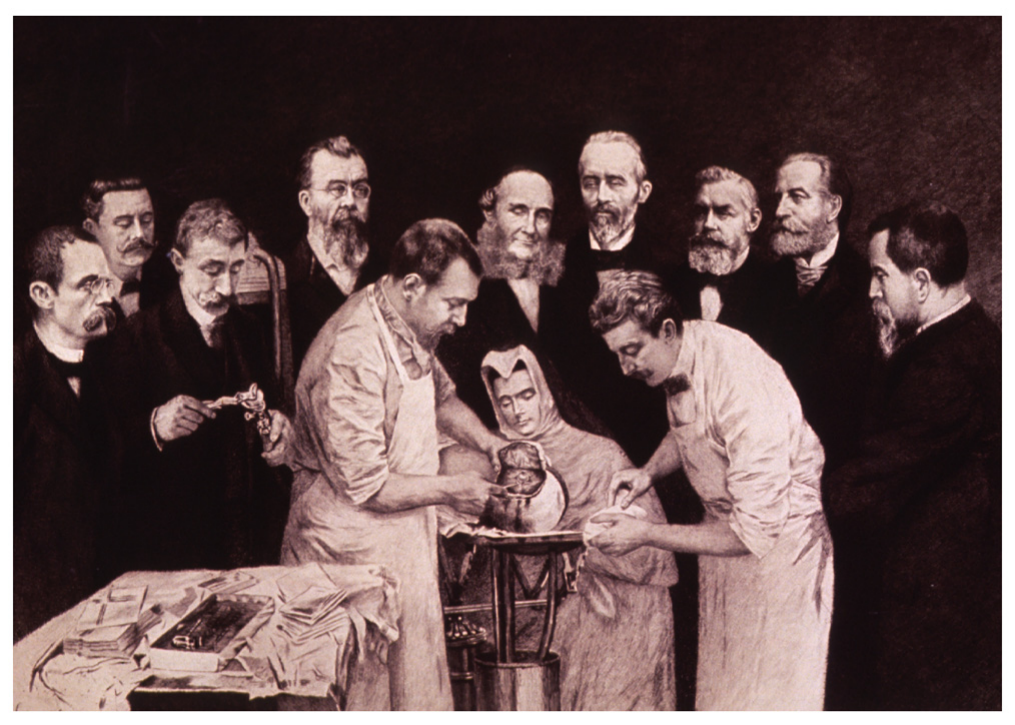
Fernand Desmoulin, Doyen performing craniectomy with aid of doctor and nurse while nine physicians observe, Paris: Porcabeuf, 1897. Engraving. US National Library of Medicine.

Franju’s knowledge of historical cinema was extensive. In 1936 he cofounded France’s most important film archive, the Cinémathèque Française, with Henri Langlois, Jean Mitry and Paul-August Harlé. After the Second World War he was appointed secretary-general of the Institut de Cinématographie Scientifique, a position he held for the next decade. For the film critic André Bazin, the French scientific cinema movement was ‘responsible for pushing back the boundaries of images acceptable to its age. It purveyed hard truths, an unbearable realism’ ^76^. Surgical and medical films had been popular in France since the turn of the century. As early as 1897 the surgeon Eugène-Louis Doyen (1859–1916) had famously commissioned films of craniectomy and hysterectomy operations at his private clinic in Paris ( Figure 3) ^77^. Over the next eight years he made more than 60 films with the cameraman Clément-Maurice, who accompanied him as a projectionist to surgical congresses across Europe ^78^. The operations followed a standard format: Doyen, sometimes with assistants, would stand facing the camera with the patient in the foreground. The film historian Thierry Lefebvre notes their ‘choreographic’ quality ^79^.

Controversy surrounding the commercial distribution of Doyen’s films suggests that there was a public appetite for medical spectacles (amongst other new scientific attractions like X-rays). Doyen himself seems to have arranged screenings in commercial cinemas, although not without opposition: a cinema in Rome was closed by authorities on one occasion ^80^. Traveling fairs had taken place across Europe since the Middle Ages, displaying human ‘oddities’ in portable booths that, by 1896, were being converted into moving picture theatres ^81^. It is here, and in the cinemas starting to open in European towns and cities, that the public would have been able to see films like Doyen’s, as well as newsreels, boxing films, travelogues, celebrations of science and industry and recreations of famous battles.

Surgical spectaculars were a theatrical phenomenon as well as a cinematic one. Between 1897 and 1962, Paris’s Theatre of the Grand Guignol shocked and delighted audiences with realistic scenes of brain surgery, enucleation, galvanism, blood transfusion, facial mutilation and decapitation ^82^. The new dramatic genre of the horror play, pioneered by playwright and essayist André de Lorde, exploited all manner of mechanical contraptions, blood-filled devices, prosthetic body parts and props (including animal eyeballs of different sizes obtained from a taxidermist). Faintings were a measure of an evening’s success, explains Mel Gordon:

During one de Lorde horror play that ended with a realistic blood transfusion, a record was set: fifteen playgoers had lost consciousness. Between sketches, the cobble-stoned alley outside the theatre was frequented by hyperventilating couples and vomiting individuals ^83^.

The face removal scene in *Les Yeux sans Visage* that was famous for making audiences faint in the 1960s is still squirm-inducing, its culminating act of violence carried out with slow precision. In his review Vas called it ‘unaesthetic’ – ‘the visual record of a skin-grafting operation’ ^84^.

**Figure 4.**
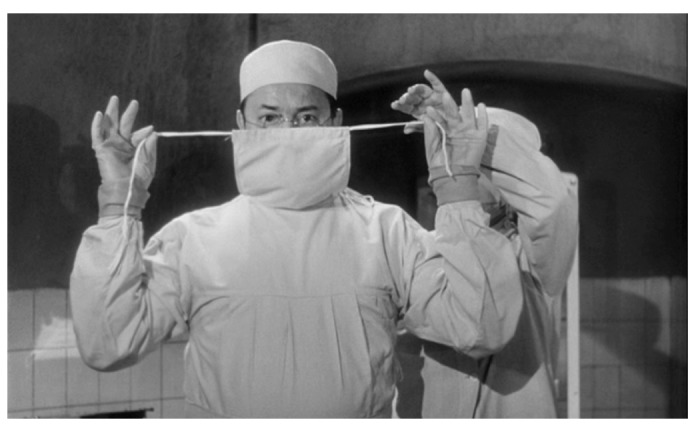
Georges Franju, *Les Yeux sans Visage*, 1959. Screenshot.

**Figure 5.**
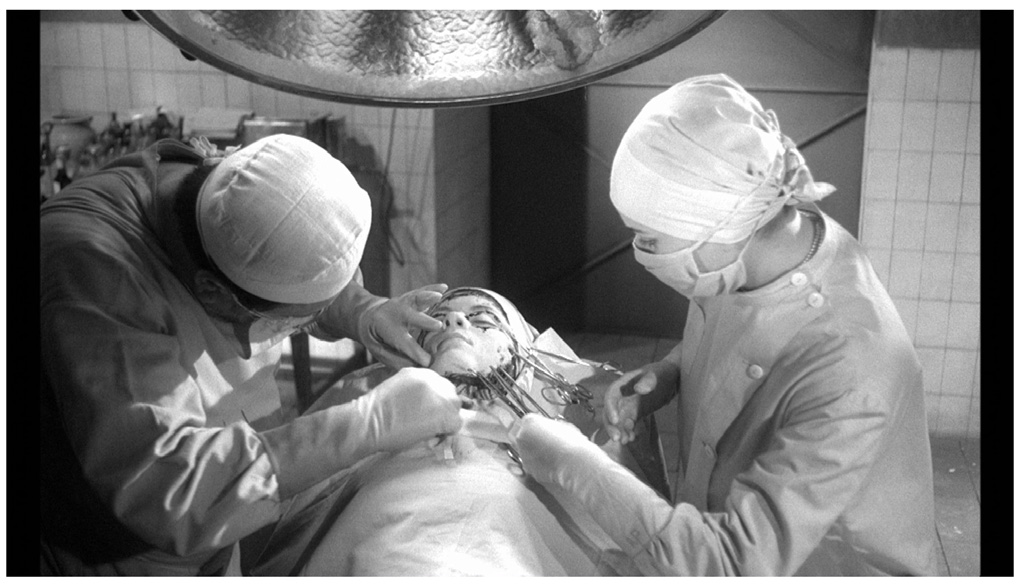
Georges Franju, *Les Yeux sans Visage*, 1959. Screenshot.

Louise has been on the prowl again and her latest acquisition is Edna Grüberg (Juliette Mayniel). Drugged and secured to a table in the secret operating theatre in Génessier’s basement, the poor girl is oblivious to her fate. The professor puts on his mask and gloves ( Figure 4). With a pencil he marks the incision lines on Edna’s face and around her eyes, his breathing audible in the silence. Beads of perspiration form on his brow as he traces the scalpel around the line. Finally, the skin is clipped and loosened with surgical clamps, then eased off ( Figure 5).

**Figure 6.**
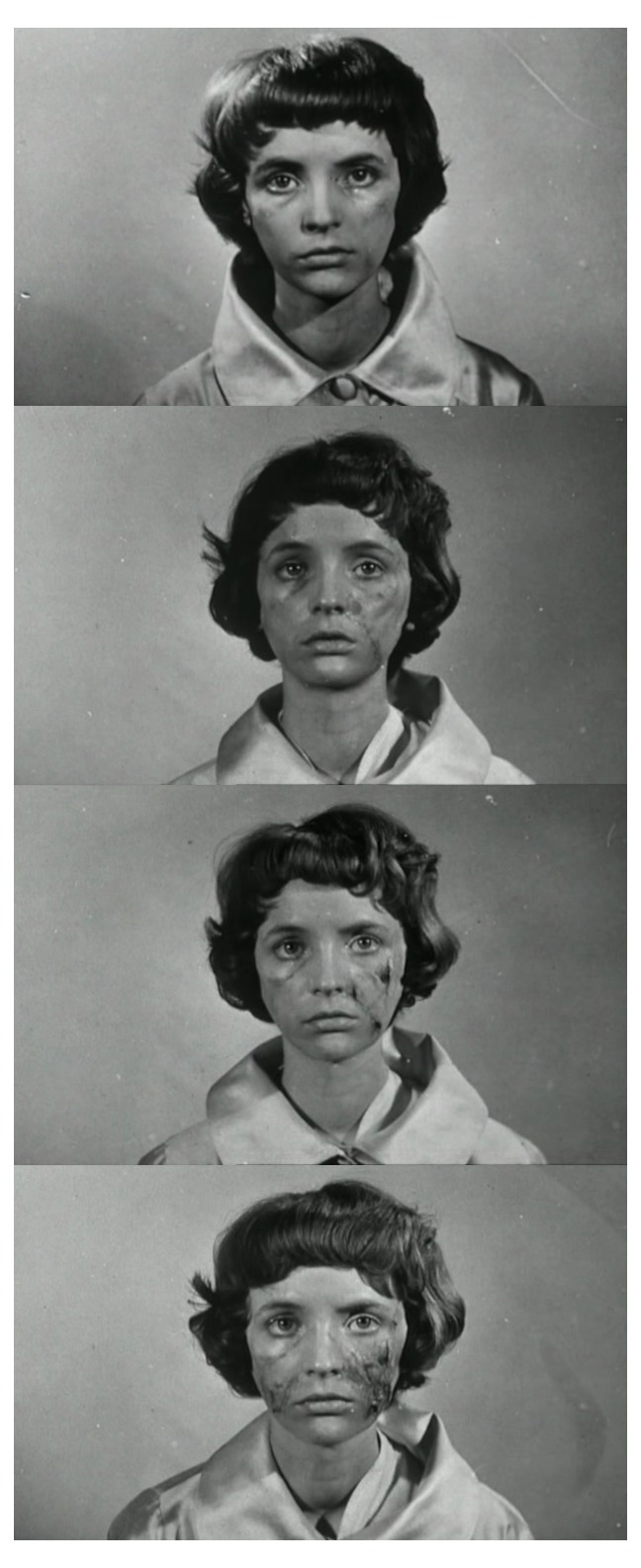
Georges Franju, *Les Yeux sans Visage*, 1959. Screenshots.

Franju’s commitment to medical realism goes beyond the operating theatre. When Christiane’s apparently successful graft begins to fail, captioned stills document the rejection of the tissue over a twenty-day period ( Figure 6). ‘February 20. A week after healing, spots of pigmentation appear’. ‘Palpation reveals small subcutaneous nodules.’ ‘On the 12th day, necrosis of the graft tissue is evident.’ ‘20th day. The first ulcerations – signs of rejection of the graft, of infection. The dead tissue must be removed.’ Visually and rhetorically, the format is precisely that of the medical case photograph. Although Christiane wears the same white satin robe, she has none of the fragile composure of earlier scenes. Taken against a neutral grey background, the photographs perform a clinical gaze that objectifies and ‘cadaverises’ its human subject ^85^. Even her eyes are vacant.

## The stylistics of horror

**Figure 7.**
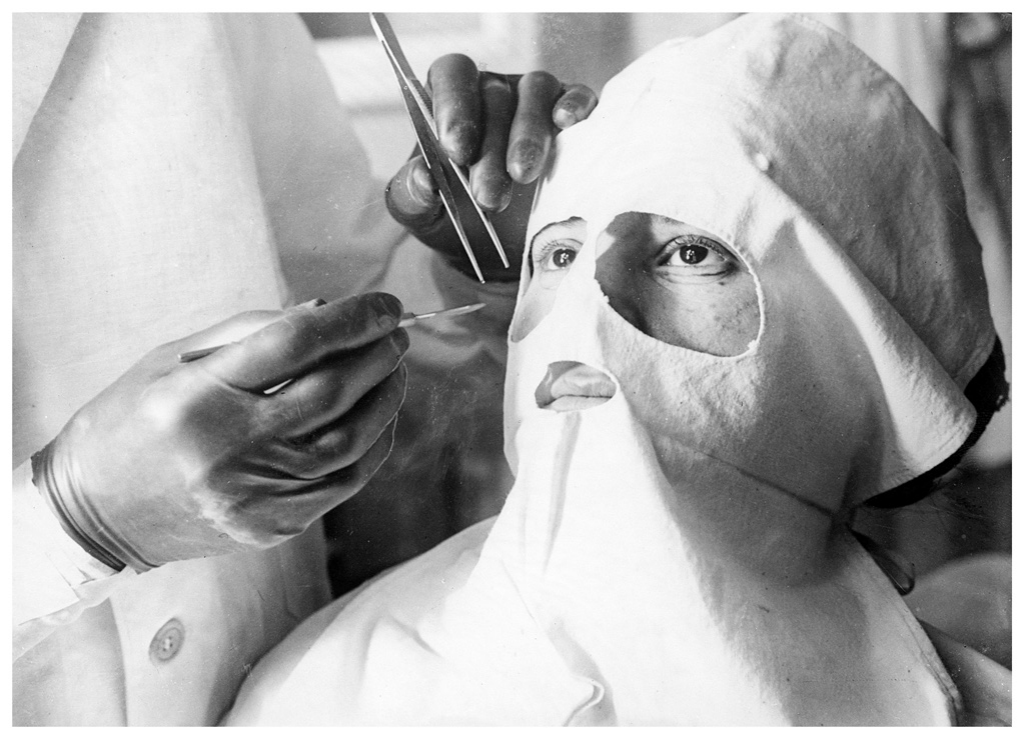
A woman undergoing 'beauty treatment' at a Berlin beauty specialist's salon in the 1930s. Unattributed photograph for Barnaby's Studios Ltd. © Mary Evans Picture Library.

The influence of Surrealism on Franju’s films has been discussed at length by Lowenstein, who focuses on the director’s affinities with the dissident Surrealist Georges Bataille ^86^. What hasn’t been explored by critics is the film’s disturbing (but very stylish) linking of femininity, horror and fashion. Violence and beauty have often converged on the aesthetic terrain of the female body in the visual culture of the twentieth century, whether in the pages of high-end fashion magazines, in horror movies, or in Surrealist art and photography ^87^. Advertisements for cosmetic beauty treatments after both world wars exploited the rhetoric and imagery of wartime reconstructive surgery with its promise of ‘new faces’ and transformed lives ^88^. The bandaged face, the beauty ‘masque’, the secluded private clinic, the white robes — all of these visual details become associated with the pursuit and enhancement of femininity ( Figure 7).

Historians have linked the medicalisation of appearance in the twentieth century to the rapid expansion of both aesthetic surgery and the global beauty industry since the 1920s ^89^. Franju’s film reflects these wider cultural realignments in medicine, fashion and beauty culture. Face transplantation, in this context, is symptomatic of a wider denaturalisation of appearance. In a 2014 interview, Scob reflected that *Eyes Without a Face* was ‘strangely premonitory.’ Today ‘there are “doctored” faces everywhere you look. … We live in a very artificial world where it seems you no longer exist when you’re no longer young. That leads to horrendous disfigurements’ ^90^. The surgical pursuit of beauty, Scob suggests, can itself be disfiguring: an idea pursued to its logical extreme in Cindy Sherman’s society portraits (2008). What the film – and Sherman’s photographs – also reveal, however, is that the ideal face is haunted by its pathological ‘other’. Appearance is both a site of desire and a source of disgust and shame. 

**Figure 8.**
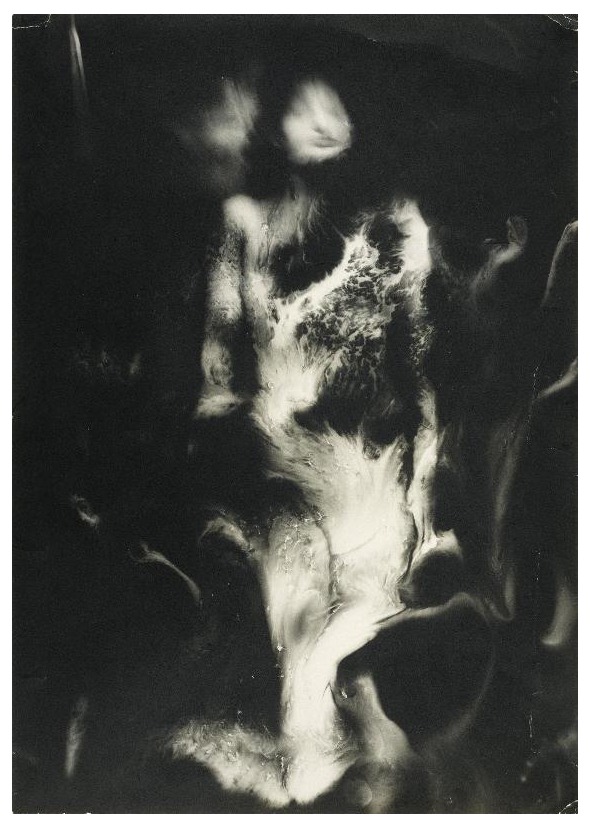
Raoul Ubac, La Nébuleuse, 1939. Gelatin silver print. Museé National d’Art Moderne, Paris. © ADAGP, Paris and DACS, London, 2018.

Surrealism provided Franju (and later Sherman) with an aesthetic of defamiliarisation; a means of redrafting ‘the most familiar of terrains’ – the female body ^91^. In an essay written to accompany the exhibition *L’Amour Fou: Photography and Surrealism* (1985), Rosalind Krauss attributed this aesthetic impulse to Bataille’s influence on the work of Man Ray, Jacques-André Boiffard, Brassaï, Raoul Ubac, Hans Bellmer, Maurice Tabard, Roger Parry, Dora Maar and other photographers associated with the Surrealist movement. Krauss identifies a series of processes aimed at distorting or disfiguring the human (typically female) body, including solarisation, photomontage, rotation, superimposition, double exposure and ‘brûlage’, which involved melting the emulsion of the photographic negative with a small burner to produce a rippled and distorted image. In Ubac’s *La Nébuleuse* (1939) for example, the ‘skin’ of the burned negative, like human skin, loses its legibility and physiognomy ( Figure 8). Bellmer’s *Dolls* similarly push the anatomically readable, living body over the threshold of the uncanny, triggering (to use the formulation Freud borrowed from Ernst Jentsch) ‘doubts whether an apparently animate being is really alive; or conversely, whether a lifeless object might not be in fact animate’ ^92^.

**Figure 9.**
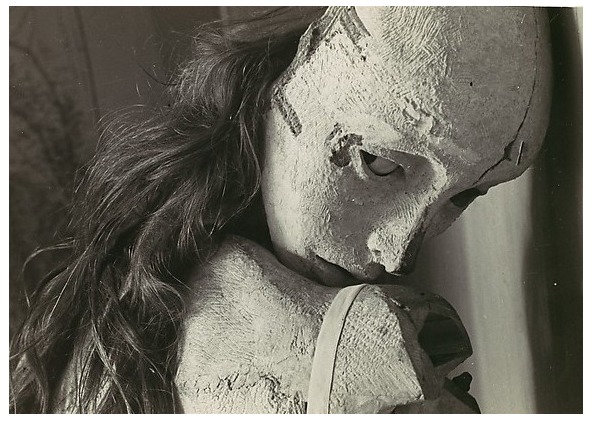
Hans Bellmer, La Poupée, ca. 1934. Gelatin silver print. Metropolitan Museum of Art: Ford Motor Company Collection, Gift of Ford Motor Company and John C. Waddell, 1987. © ADAGP, Paris and DACS, London, 2018.

Bellmer’s dismembered and grotesquely reassembled doll-fetishes present a disturbing visual parallel to Génessier’s female ‘experiments’ ( Figure 9). In his 1906 essay on ‘The Psychology of the Uncanny’, Jentsch had referred to the unsettling impression made by waxwork figures, mechanical dolls and automata: almost-human objects that reappear in subsequent discussions of the uncanny, including Freud’s. The legacy of Freud’s 1919 essay, ‘Das Unheimliche’ can be traced through Surrealism to contemporary art and fashion ^93^. Within this creative field, the mannequin is queen of the muses – the ‘silent partner’ of painters, sculptors, fashion designers, photographers and filmmakers from Georges Méliès and Giorgio De Chirico to Man Ray, Oskar Kokoschka, Paul Delvaux and of course Hans Bellmer ^94^.

**Figure 10.**
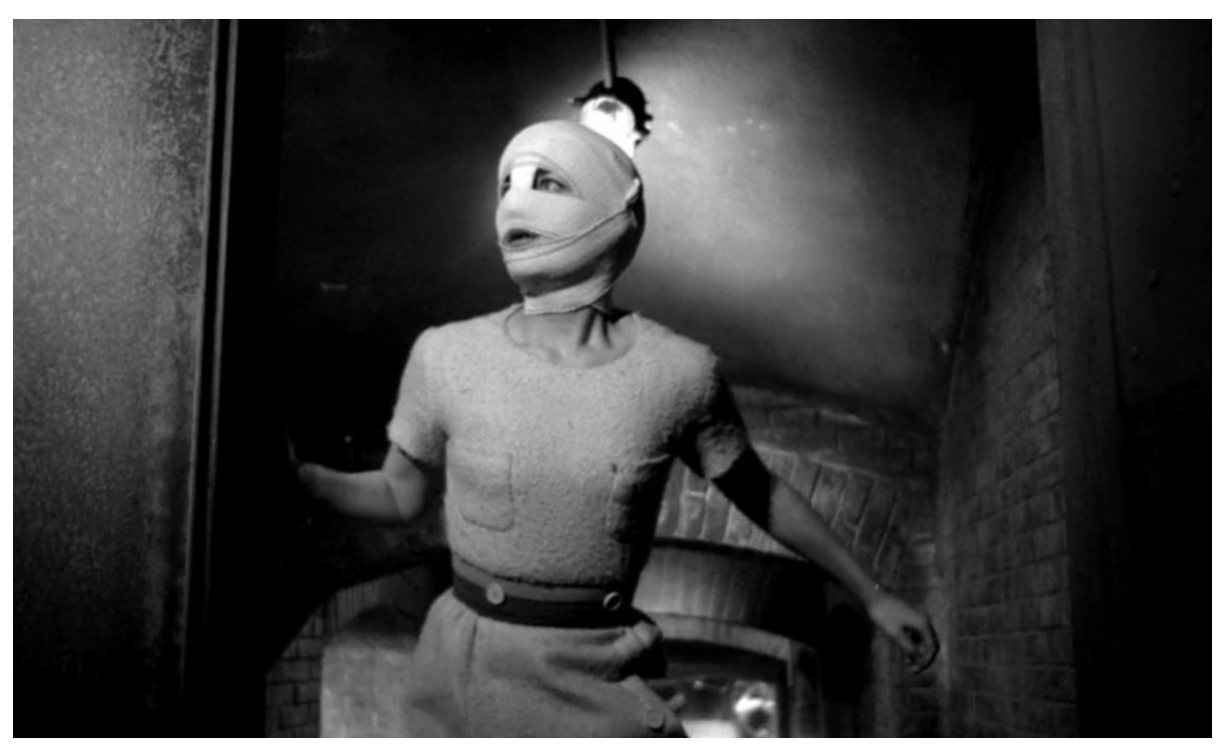
Georges Franju, *Les Yeux sans Visage*, 1959. Screenshot.

Franju ‘saw himself as a puppeteer and his actors as puppets’, comments Queysanne ^95^. He had no interest in psychological naturalism, and didn’t rehearse scenes prior to shooting, yet he was obsessive about how his actors stood, their position, pose and silhouette. ‘He wanted precise gesture. You had to hold the glass a certain way. He wanted it framed and frameable … a bit like the way a painter gets his model to strike a pose,’ says Scob ^96^. When we see Edna after the fateful operation she is being kept in a locked cell, her head wrapped in bandages leaving only her eyes and mouth visible. ‘Look after her, feed her,’ says Génessier to Louise. Edna makes one attempt to escape after knocking Louise unconscious with a bottle ( Figure 10). Hearing Génessier’s car in the driveway, she flees upstairs and hides in an attic room full of dresses, dolls, a harp: the ghostly detritus of bourgeois family life. Realising she has no other options, Edna jumps out of the open window. We see her lifeless body on the pavement below, followed by a close-up of her wide-open eyes.

**Figure 11.**
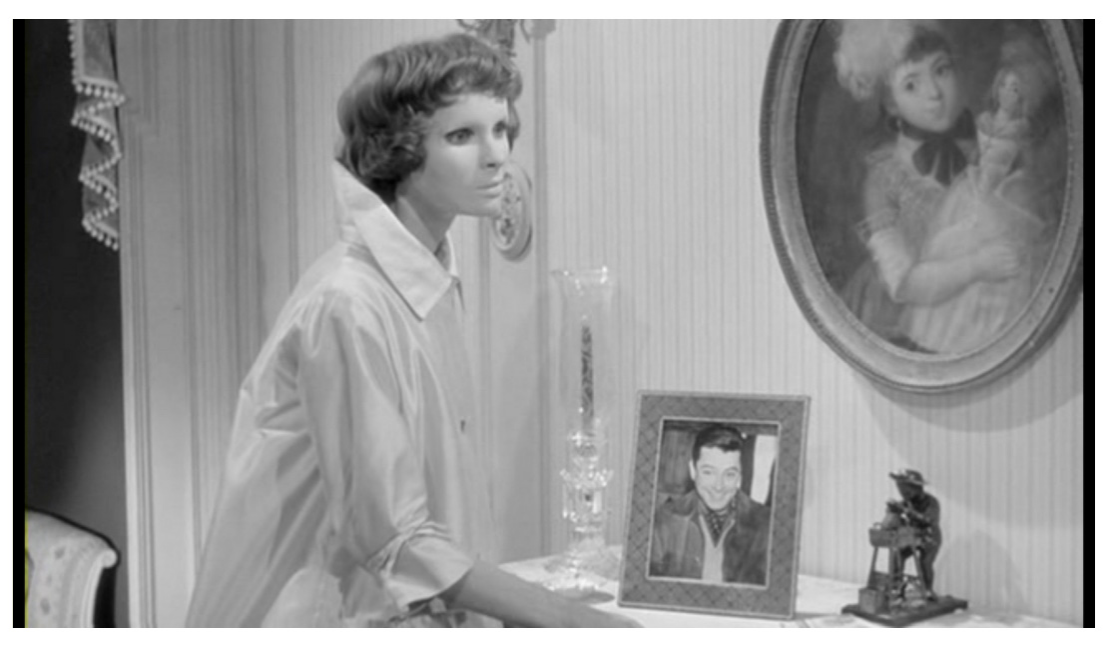
Georges Franju, *Les Yeux sans Visage*, 1959. Screenshot.

Christiane is Edna’s spectral double; a cross between a life-size doll and a shop mannequin ( Figure 11). The American film critic Pauline Kael attributed her costumes to Givenchy ^97^. For most of the film she wears a high-collared, full-length white housecoat that stands away from her body like satin architecture. The mannequins that mutely inhabit Surrealist photographs are rarely clothed (certainly never as demurely as this), but Christiane is no more human than Raoul Ubac’s *Tête du Mannequin d’André Masson* (1938, Figure 12). Franju liked her ethereal quality: she ‘looked like somebody who walked without touching the ground’ ^98^.

A fascination with masks, and the trope of the made-up face as mask, runs through avant-garde modernism as well as twentieth-century fashion photography and cosmetics advertising. Christiane’s mask – which Dilys Powell in her review called ‘modish’ – is not so different from the whiteout effect of 1950s make-up, with its opaque layers of liquid foundation and loose powder ( Figure 13) ^99^. For *Les Yeux sans Visage* Scob had three masks, each cast in plastic from a single plaster mould of her face. One was easy to remove, but the others were ‘like a portable jail,’ worn directly on the skin and applied in a makeup session at the start of each day. Because the mask was so thin, it moved if she tried to talk, so she dubbed herself in post-production. ‘I was like an object,’ Scob recalls. ‘A face is a language, and suddenly that language had been taken away from me’ ^100^.

**Figure 12.**
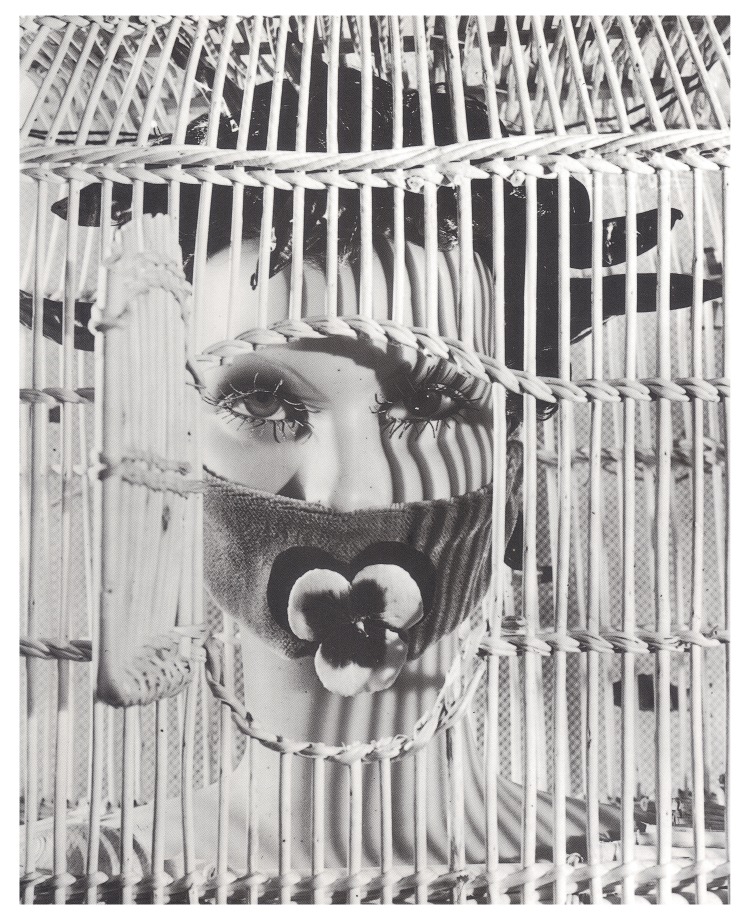
Raoul Ubac, Tête du Mannequin d’André Masson, 1938. Galerie Adrien Maeght, Paris. © ADAGP, Paris and DACS, London, 2018.

**Figure 13.**
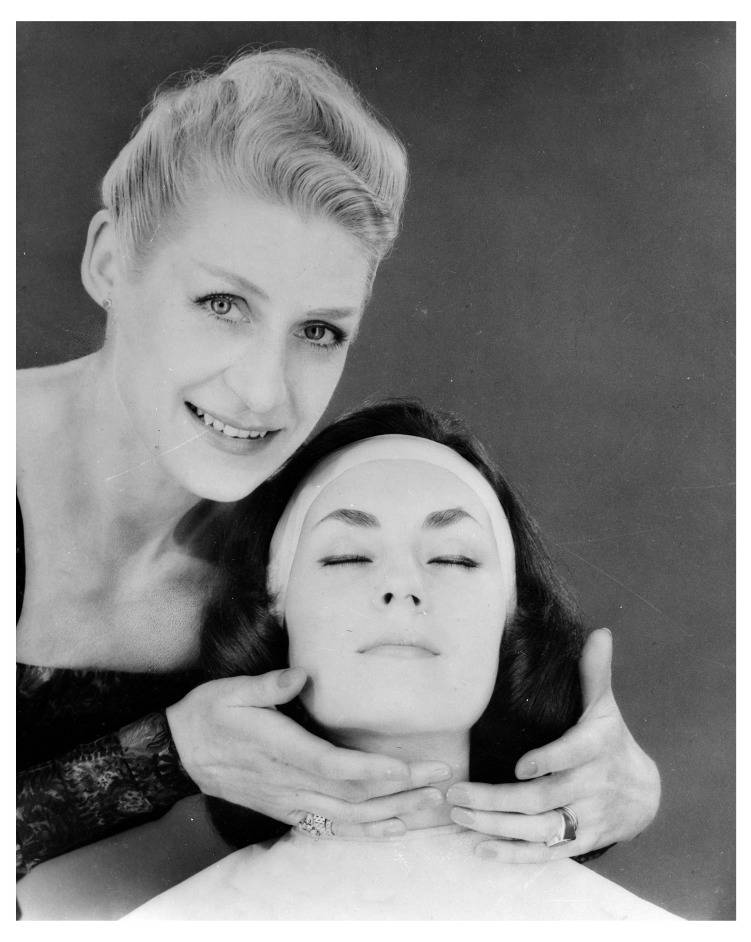
Advertisement for Elizabeth Arden cosmetics (Anne Wilkie and Geraldine Hill), 1959. Photograph by Colin Sherborne. ©The Colin Sherborne Collection/Mary Evans Picture Library.

## Conclusion

**Figure 14.**
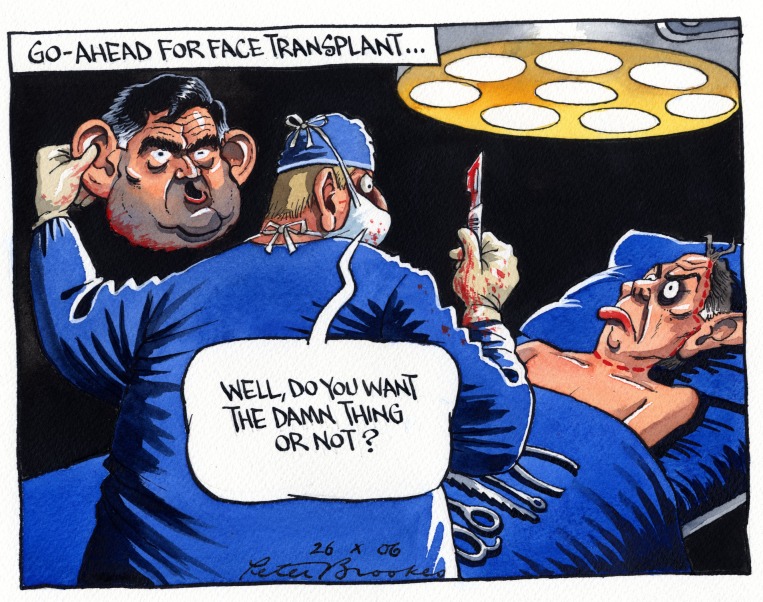
Peter Brookes, ‘Go-ahead for face transplant …’ *The Times*, October 26, 2006. © The Times/News Licensing.

It is surely no coincidence that face transplantation emerges as a cinematic event at a moment in the twentieth century when the medicalisation and commodification of female appearance had become mainstream. The medical literature rarely references this history (and then only to insist that face transplantation is not an aesthetic procedure), but early press reports of Dinoire’s operation often made the connection ^101^. Placing face transplantation within the history of the ‘extreme makeover’ might sound frivolous, but it highlights the extent to which the discourse of appearance management has entered popular culture. In *Face/Off*, John Woo’s 1997 action thriller in which Nicholas Cage and John Travolta swap faces and identities, face transplantation is presented as high-tech cosmetic surgery. Manohla Dargis, writing for *Sight & Sound*, noted the irony of ‘a Hollywood action movie that hinges on a facelift’. By the 1990s, plastic surgery had become ‘one of the most socially acceptable acts of violence you can commit in Hollywood.’ ^102^ Political cartoons occasionally exploit these associations as well: a 2006 drawing by Peter Brookes depicts British Prime Minister Tony Blair being offered the face of Gordon Brown, his soon-to-be successor ( Figure 14). ‘Well, do you want the damn thing or not?’ asks the surgeon, beneath the caption ‘Go ahead for face transplant’. A Kenneth Mahood cartoon in the *Daily Mail* earlier the same year features Blair with a big yellow smiley face and the caption: ’Good Heavens, don't tell me the PM has decided to have the world's first full face transplant!' ^103^. In both examples, face transplantation is a metaphor for dissimulation.

The face transplant debate has been ‘poisoned by a fantasy view of what face transplant is,’ wrote Lantieri in 2009, ‘especially after movies like *Face/Off* … or *Eyes without a Face* … which showed face transplant as the shifting of faces, rather than the reconstruction of faces’ ^104^. In fact one of the surprising things about Franju’s film is that the ‘shifting of faces’ and identities is not developed as a theme. Discussing the practical implications of Christiane’s staged funeral and altered appearance, Génessier points out matter-of-factly that she cannot re-enter the world as herself. The deception will have to continue. False papers, a long holiday perhaps: ‘A new face, a new identity. It will be fun.’ Unlike the face swap conceit in *Face/Off*, it is not implied that Christiane now looks like Edna. Rather, it seems the victims are chosen because they look passably like her: their faces will enable her restoration to the world of the living. The physiognomy of the ‘new’ Christiane is indistinguishable from the original: a detail that distances the film from the trope of surgical transformation in Hollywood cinema of the 1920s onwards ^105^. In *Eyes without a Face* the only difference between the old Christiane and the new is that her mask has become flesh, like the milk-white ivory statue of Galatea that Venus brings to life for Pygmalion in Ovid’s *Metamorphosis*. As Christiane sits at dinner in a gown resplendent with flowers, Louise remarks ‘You’re more beautiful than ever. Now there’s something angelic about you.’ ‘Angelic, I wouldn’t know,’ the young woman answers. ‘When I look into a mirror, I feel I’m seeing someone who looks like me, returning from far away.’

This article began with two very different approaches to the cultural history of the face transplant: one aimed at justifying and naturalising transplant surgery, the other an attempt to problematize it. Both versions fail to take account of the myriad ways in which medical practices and paradigms enter popular culture and the contexts that invest old stories with new meaning. As face transplantation acquires its own history – or possibly multiple, contested histories – it is important that these contexts are not forgotten, and that the fault lines in opinion are registered. It seems likely that one of the significant variables will be gender, as Fay Bound Alberti has suggested: both in terms of how face transplantation is represented, and how it is experienced by those personally involved ^106^. Another factor will be how ‘identity’ is understood: a term that continues to provoke confusion and disagreement in the medical and ethical literature ^107^.

I have approached face transplantation as a twentieth-century phenomenon, not an idea that has been around for millennia. Beginning with Franju’s *Les Yeux sans Visage*, the cinematic pre-history of the face transplant establishes stylistic, rhetorical and performative conventions that continue (for better or worse) to influence media representations and academic debate about the procedure. At the same time, Franju’s sources – the scientific cinema movement, Grand Guignol, Surrealist film and photography, even the popular imagery of fashion and beauty – point to the many unexplored contexts in which the modern surgical imaginary has taken shape. All of these practices played a part in redefining the human body in the twentieth century, as did public debates about organ harvesting and transplantation and the prospect of a ‘denatured’ future. No longer a uniquely individual attribute – a material signifier of personhood – the removable, transplantable face becomes an ambiguous entity. Not quite a ‘spare part’ perhaps, but something that can be donated or stolen, tolerated or rejected, an intimate gift and a risky investment.

## Data availability

All data underlying the results are available as part of the article and no additional source data are required.

## Footnotes

^1^ Simon Hattenstone, ‘Face Saver’, *The Guardian*, 2007 < https://www.theguardian.com/lifeandstyle/2007/nov/10/healthandwellbeing.features1> [accessed 26 April 2017].

^2^ Hattenstone.

^3^ Leslie Fiedler, ‘Why Organ Transplant Programs Do Not Succeed’, in *Organ Transplantation: Meanings and Realities*, ed. by Stuart J. Youngner, Renee C. Fox, and Laurence J. O’Connell, 1996, pp. 56–65 (p. 57).

^4^ Fiedler, p. 61.

^5^ Maria Siemionow, *Transplanting a Face: Notes on a Life in Medicine* (Cleveland, OH: Cleveland Clinic Press, 2008), p. 148.

^6^ Francois Petit, Antoine Paraskevas, and Laurent Lantieri, ‘A Surgeons’ Perspective on the Ethics of Face Transplantation’, *The American Journal of Bioethics*, 4.3 (2004), 14–16 (p. 15).

^7^ Austin, Naomi, *BBC Horizon, The World’s First Face Transplant*, 2006.

^8^ Fiedler, p. 63.

^9^ Susan E. Lederer, *Flesh and Blood: Organ Transplantation and Blood Transfusion in 20th-Century America* (Oxford: Oxford University Press, 2008), p. ix.

^10^ Lederer, p. 211.

^11^ Laurent Lantieri, ‘Face Transplant: A Paradigm Change in Facial Reconstruction’, *Journal of Craniofacial Surgery*, 23.1 (2012), 250–53 (p. 250).

^12^ Lantieri, p. 250.

^13^ Siemionow, p. 26.

^14^ Roy Yorke Calne, ‘Transplant | Surgery’, *Encyclopedia Britannica* < https://www.britannica.com/topic/transplant-surgery> [accessed 23 May 2017].

^15^ James D. Hardy, ‘Organ Transplantation’, in *An Introduction to the History of General Surgery*, ed. by Richard Hardaway Meade (Philadelphia: W. B. Saunders, 1968), pp. 364–74 (p. 364).

^16^ *Transplantation*, ed. by John S. Najarian and Richard L. Simmons (Philadelphia: Lea & Febiger, 1972), p. vii.

^17^ Thomas Schlich, ‘How Gods and Saints Became Transplant Surgeons: The Scientific Article as a Model for the Writing of History’, *History of Science*, 33.3 (1995), 311–31 (p. 312).

^18^ Schlich, p. 313.

^19^ Schlich, pp. 314, 317.

^20^ Schlich, p. 322.

^21^ Bruce W. Conolly and Mario Benanzio, ‘Cosmas and Damian Revisited’, in *Hand Transplantation*, ed. by Marco Lanzetta and Jean-Michel Dubernard (Springer, 2007), pp. 3–10.

^22^ Leonard Barkan, ‘Cosmas and Damian: Of Medicine, Miracles, and the Economies of the Body’, in *Organ Transplantation: Meanings and Realities*, ed. by Stuart J. Youngner (Madison: University of Wisconsin Press, 1996), pp. 221–51 (p. 224).

^23^ Barkan, p. 225.

^24^ Jacopus de Voragine, ‘Saints Cosmas and Damian’, in *The Golden Legend: Readings on the Saints*, trans. by William Granger Ryan, 2 vols (Princeton: Princeton University Press, 1993), II, 196–98.

^25^ John Farndon, *From Laughing Gas to Face Transplants: Discovering Transplant Surgery*, Chain Reactions (Lewes: Heinemann Library, 2006), p. 5.

^26^ Barkan, p. 228.

^27^ Barkan, p. 228.

^28^ Barkan, p. 234.

^29^ Conolly and Benanzio, p. 10.

^30^ Wendy Doniger, ‘The Mythology of the Face-Lift’, *Social Research*, 2000, 99–125 (pp. 101–3).

^31^ Annie Dillard, *Pilgrim at Tinker Creek* (New York: Bantam Books, 1975), p. 273.

^32^ Wendy Doniger, ‘Transplanting Myths of Organ Transplants’, in *Organ Transplantation: Meanings and Realities*, ed. by Stuart J. Youngner, Renee C. Fox, and Laurence J. O’Connell (Madison: University of Wisconsin Press, 1996), pp. 194–220 (pp. 200–201).

^33^ Doniger, ‘Transplanting Myths of Organ Transplants’, p. 201.

^34^ Quoted in Sander L. Gilman, *Making the Body Beautiful: A Cultural History of Aesthetic Surgery* (Princeton: Princeton University Press, 1999), p. 68.

^35^ Martha Teach Gnudi and Jerome Pierce Webster, *The Life and Times of Gaspare Tagliacozzi* (New York: Herbert Reichner, 1950), fig. 41.

^36^ Samuel Butler, *Hudibras, Parts I and II and Selected Other Writings*, ed. by John Wilders and Hugh de Quehen (Oxford: Clarendon Press, 1973), pp. 281–83.

^37^ Gnudi and Webster, pp. 282–85.

^38^ Emily Cock, ‘“Off Dropped the Sympathetic Snout”: Shame, Sympathy, and Plastic Surgery at the Beginning of the Long Eighteenth Century’, in *Passions, Sympathy and Print Culture*, ed. by Heather Kerr, David Lemmings, and Robert Phiddian (Houndmills: Palgrave Macmillan, 2016) < https://dx.doi.org/10.1057/9781137455413>.

^39^ Marc Lafrance, ‘“She Exists within Me”: Subjectivity, Embodiment and the World’s First Facial Transplant’, in *Abjectly Boundless: Boundaries, Bodies and Health Work*, ed. by Trudy Rudge and Dave Holmes (Farnham: Ashgate, 2010), pp. 147–61.

^40^ Radu Florescu, *In Search of Frankenstein* (London: Robson, 1996), pp. 276–82. The filmography covers the years from 1910 to 1994.

^41^ Fiedler, p. 63.

^42^ Fiedler, p. 62.

^43^ Fiedler, p. 61.

^44^ Fiedler, p. 61.

^45^ Fiedler, p. 64.

^46^ Fiedler, p. 60.

^47^ Carla Bluhm and Nathan Clendenin, *Someone Else’s Face in the Mirror: Identity and the New Science of Face Transplants* (Westport, CT: Praeger, 2009), p. xiv.

^48^ Lederer, p. 210.

^49^ Arthur L. Caplan, *If I Were a Rich Man Could I Buy a Pancreas?* (Bloomington: Indiana University Press, 1994).

^50^ Lederer, p. 211.

^51^ Robert Vas, ‘Review of Eyes Without a Face’, *Sight & Sound*, 29.2 (1960), 92 (p. 93).

^52^ Roger Luckhurst, ‘Science Fiction and Cultural History’, *Science Fiction Studies*, 37.1 (2010), 3–15 (p. 13).

^53^ Organ transplantation timelines usually begin in 1954, with the first successful kidney transplant between identical twins. Other landmarks include the first lung (1963), pancreas (1966), liver (1967) and heart (1967) transplants.

^54^ Facial reconstruction makes an early appearance in Hollywood cinema: Kember notes that the American Film Institute Catalogue lists 16 movies with plastic surgery themes between 1922 and 1930. Unlike horror cinema however, films like *Skin Deep* (1922), *Back to Life* (1925) and *Face Value* (1927) use plastic surgery as a vehicle for ‘ethical transformation’ and social restitution, often following the return to domestic and civilian life of facially wounded First World War veterans. Joe Kember, ‘Face Value: The Rhetoric of Facial Disfigurement in American Film and Popular Culture, 1917–1927,’ *Journal of War and Culture Studies*, 10.1 (2017), 43–65 (pp. 51–53, 63n.2).

^55^ Phil Hardy, *Encyclopedia of Horror Movies* (New York: Harper & Row, 1986), p. 125.

^56^ Raymond Durgnat, *Franju* (London: Studio Vista, 1967), p. 79; Kate Ince, *Georges Franju* (Manchester: Manchester University Press, 2005), p. 50; David Taylor, ‘Masks, Masques, and the Illusion of Reality: The Films of Georges Franju’, in *Shock Xpress 2*, ed. by Stefan Jaworzyn (London: Titan Books, 1994), pp. 6–16 (p. 7).

^57^ Dilys Powell, ‘Savage, Sombre and Revolting’, *Sunday Times* (London, England, 6 September 1959), p. 27.

^58^ Jon Turney, *Frankenstein’s Footsteps: Science, Genetics and Popular Culture* (New Haven: Yale University Press, 2000).

^59^ Theodore I Malinin, *Surgery and Life: The Extraordinary Career of Alexis Carrel* (New York: Harcourt Brace Jovanovich, 1979), pp. 46–47.

^60^ Carl Snyder, ‘Carrel: Mender of Men’, *Collier’s Weekly*, 16 November 1912, 12–13 (p. 13).

^61^ Guy Austin, ‘Biological Dystopias: The Body in Contemporary French Horror Cinema’, *L’Esprit Créateur*, 52.2 (2012), 99–113 (p. 104).

^62^ Jean Rostand, *Can Man Be Modified?*, trans. by Jonathan Griffin (London: Secker & Warburg, 1959), pp. 34, 39.

^63^ Quoted in Rostand, pp. 65–66.

^64^ Rostand, p. 67.

^65^ Durgnat, p. 84.

^66^ Hardy (1968, p. 366) notes that the ‘previously somewhat imprecise terms of *homograft* and *heterograft* have been dropped by many workers and replaced by *allograft* [a graft between two genetically distinct members of the same species] and *xenograft* [between different species]’.

^67^ Durgnat, p. 85.

^68^ Joan Hawkins, *Cutting Edge: Art-Horror and the Horrific Avant-Garde* (Minneapolis: University of Minnesota Press, 2000), p. 68.

^69^ Adam Lowenstein, *Shocking Representation: Historical Trauma, National Cinema, and the Modern Horror Film* (New York: Columbia University Press, 2005), p. 42.

^70^ Curtis Bowman, ‘A Film without Politics: Georges Franju’s Les Yeux sans Visage’, *Kinoeye*, 2.13 (2002) < http://www.kinoeye.org/02/13/bowman13.php> [accessed 15 June 2017].

^71^ Lowenstein, pp. 1–2.

^72^ Ince, p. 106.

^73^ Durgnat, p. 28.

^74^ Ince, pp. 107–8.

^75^ Pierre-Henri Gibert, *Les Fleurs Maladives de Georges Franju*, 2009.

^76^ Quoted in Ince, p. 110.

^77^ Virgilio Tosi, *Cinema Before Cinema: The Origins of Scientific Cinematography* (London: British Universities Film & Video Council, 2005), p. 165.

^78^ Tiago Baptista, ‘Il Faut Voir Le Maître: A Recent Restoration of Surgical Films by E.L. Doyen (1859-1916)’, *Journal of Film Preservation*, 70 (2005), 42–50 (p. 43).

^79^ Quoted in Baptista, p. 44.

^80^ Tosi, p. 168.

^81^ Richard Abel, *Encyclopedia of Early Cinema* (London: Routledge, 2005), p. 227.

^82^ Mel Gordon, *The Grand Guignol: Theatre of Fear and Terror* (New York: Amok Press, 1988), pp. 2, 76–81.

^83^ Gordon, pp. 27–28.

^84^ Vas, p. 92.

^85^ Foucault traces the emergence of a ‘clinical’ gaze in *Naissance de la Clinique* (1963), published in English as *The Birth of the Clinic: An Archaeology of Medical Perception*, trans. A. M. Sheridan (London: Routledge, 1989). The reference to the ‘absolute eye that cadaverizes life’ is on p. 204.

^86^ Lowenstein, pp. 19–20.

^87^ On the common ground between fashion photography, cosmetics advertising and Surrealism see: Lucy Moyse, ‘Manifestations of Violence and Trauma within Interwar Fashion, in London, Paris and New York’ (unpublished PhD thesis, The Courtauld Institute of Art, 2017).

^88^ Beth Haiken, ‘Plastic Surgery and American Beauty at 1921’, *Bulletin of the History of Medicine*, 68.3 (1994), 429–53; Elizabeth Haiken, *Venus Envy: A History of Cosmetic Surgery* (Baltimore: Johns Hopkins University Press, 1997), pp. 132–33.

^89^ Elizabeth Haiken; Geoffrey Jones, *Beauty Imagined: A History of the Global Beauty Industry* (Oxford: Oxford University Press, 2010); Kathy Peiss, *Hope in a Jar: The Making of America’s Beauty Culture* (Philadelphia: University of Pennsylvania Press, 2011).

^90^ LP Hugo, *For Her Eyes Only: An Interview with Edith Scob* (BFI, 2014).

^91^ Rosalind Krauss, ‘Corpus Delicti’, in *L’Amour Fou: Photography and Surrealism*, ed. by Rosalind Krauss and Jane Livingston (New York: Abbeville Press, 1985), p. 60.

^92^ Sigmund Freud, ‘The Uncanny’, in *The Standard Edition of the Complete Psychological Works of Sigmund Freud*, ed. by James Strachey, 24 vols (London: Hogarth Press), 17 (1917-1919), 217–52 (p. 225).

^93^ Mike Kelley and John C. Welchman, *Mike Kelley: The Uncanny* (König / Tate Liverpool, 2004); Richard Martin, *Fashion and Surrealism* (New York: Rizzoli, 1987); Nicholas Royle, *The Uncanny* (Manchester: Manchester University Press, 2003); Ghislaine Wood, *The Surreal Body: Fetish and Fashion* (London: Victoria & Albert Museum, 2007).

^94^ *Silent Partners: Artist and Mannequin from Function to Fetish* was the title of a 2014-15 exhibition at the Fitzwilliam Museum, Cambridge. **

^95^ Gibert.

^96^ Gibert.

^97^ Pauline Kael, *I Lost It at the Movies: Film Writings, 1954-1965* (Marion Boyars, 1994), p. 7; Tim Lucas, ‘Skin Scare’, *Sight & Sound*, 15.1 (2005), 75.

^98^ Hugo.

^99^ Powell.

^100^ Hugo.

^101^ Ruth La Ferla and Natasha Singer, ‘The Face of the Future’, *The New York Times*, 16 December 2005 < http://www.nytimes.com/learning/teachers/featured_articles/20051216friday.html> [accessed 21 March 2017].

^102^ Manohla Dargis, ‘Do You like John Woo?’, *Sight & Sound*, 7.9 (1997), 10–12 (p. 10).

^103^ Kenneth Mahood, *Daily Mail* (London, 19 June 2006). British Cartoon Archive, University of Kent.

^104^ Powell, p. 27.

^105^ Kember, pp. 51-53.

^106^ Fay Bound Alberti, ‘From *Face/Off* to the Face Race: The Case of Isabelle Dinoire and the Future of the Face Transplant’, *Medical Humanities*, 2016, medhum-2016-011113 < https://doi.org/10.1136/medhum-2016-011113>.

^107^ George J. Agich and Maria Siemionow, ‘Until They Have Faces: The Ethics of Facial Allograft Transplantation’, *Journal of Medical Ethics*, 31.12 (2005), 707–9; Françoise Baylis, ‘A Face Is Not Just like a Hand: Pace Barker’, *The American Journal of Bioethics*, 4.3 (2004), 30–32; Alex Clarke and others, ‘Attitudes to Face Transplantation: Results of a Public Engagement Exercise at the Royal Society Summer Science Exhibition’, *Journal of Burn Care & Research*, 27.3 (2006), 394–98; Lafrance; Ajay Modgil, ‘Identity Transfer and Identity Restoration in Facial Allotransplantation’, *Eplasty*, 11 (2011); Jessica J. Prior and Orly Klein, ‘A Qualitative Analysis of Attitudes to Face Transplants: Contrasting Views of the General Public and Medical Professionals’, *Psychology & Health*, 26.12 (2011), 1589–1605; John A. Robertson, ‘Face Transplants: Enriching the Debate’, *The American Journal of Bioethics*, 4.3 (2004), 32–33; Nichola Rumsey, ‘Psychological Aspects of Face Transplantation: Read the Small Print Carefully’, *The American Journal of Bioethics*, 4.3 (2004), 22–25; B. E. White and I. Brassington, ‘Facial Allograft Transplants: Where’s the Catch?’, *Journal of Medical Ethics*, 34.10 (2008), 723–26.
