# Supplementary material for: Theatres of surgery: The cultural pre-history of the face transplant
Source: Wellcome Open Res. 2018 May 8;3:54. [Version 1] doi: 10.12688/wellcomeopenres.14558.1 (PMC6051199; doi:10.12688/wellcomeopenres.14558.1)
Supplement: Peer review history

# Peer review: Referee response for version 1

**DOI:** 10.21956/wellcomeopenres.15846.r33108

**Published:** 2018-07-11

**Authors:** Joe Kember

**Affiliations:** 1 University of Exeter, Exeter , UK

Across the past twenty years, the study of the face has become a prime site for cultural and philosophical investigations centred on the human body and especially on notions of empathy and emotional response. In film studies (my own field) for example, a persistent post-Deleuzean trend has resulted in the development of new studies of the face in cinema which, according to Thomas Elsaesser and Malte Hagener ^1^, can at this point even be said to have developed their own "research areas" (p. 69), closely affiliated with both phenomenological and cognitive models of affect. In this context, consideration of the idea of face transplantation, both as a medical reality first realised in 2005 and as a cultural trope that has persisted across several centuries, seems an enormously productive avenue of enquiry, not least in film, where the wearing of faces has a substantial history of its own, inside and outside of the horror genres. Alongside an ancient lineage of ideas connected to disguise and masking, the face transplant presents a complex knot of ethical, emotional, and corporeal concerns, many of which appear on the surface of contemporary press reports concerning this procedure, but which have remained curiously under-examined in all types of scholarly work.

 In this article, Biernoff does not refer to much of this scholarship in film studies, nor to studies of affect, but draws instead from a broadly-conceived cultural history, stretching back many centuries and across trans-national and trans-cultural boundaries, in order to develop a nuanced and multifaceted argument. The strength of this approach, in my view, is that it allows for a diverse account of several of the cultural readings that have gathered around the idea of the face transplant and, in particular, a useful analysis of the written work of the new generation of surgeons, who have now begun to make this a more common procedure. Finding that such individuals borrow freely not only from historical but from mythological sources in order to trace a genealogy of their surgical practices, Biernoff argues that this type of discourse largely serves to legitimise the practice of transplantation; however, all along there have also been other discourses at work. For me, the most significant aspect of Biernoff’s argument lies in the way she resists structuring her argument around an essentialised divine healer/mad scientist binary, instead suggesting that surgical innovations have had a far more fluid discursive role across time. Leslie Fiedler’s pioneering work ^2^ is a touchstone for much of this argument, and in an extended piece I would also have liked to see some engagement with other works, such as his 1979 book, *Freaks: Myths and Legends of the Secret Self*, a volume which surely delivers a more varied diet of tropes of troubled selfhood across a wider range of representations.

 But Biernoff is more interested here in detailing culturally specific resonances for face transplantation, and correspondingly, the main thrust of the analysis is dedicated to a reading of a single 1959 text, Georges Franju’s classic *Les Yeux sans Visage*. This film is well chosen: while much commentary has addressed the central, remarkably gory, scene of the transplant itself –  a clear evocation of gothic as well as scientific progress tropes – other scenes also revolve obsessively around faces, faces that are hidden, masked, bandaged, or marked, human and animal faces, faces distorted with terror or entirely inexpressive. In a medium which has evolved primarily to develop the potential of the face as a site of communication and empathy, Franju’s film is remarkable for its exploitation of the face as a property with other capacities. Above all, perhaps, and in common with numerous examples of the horror genres the film helped to renew and inaugurate, *Les Yeux sans Visage* leaves a lasting memory of faces that serve primarily as objects: items that can be divorced from their internal moorings, literally and figuratively, and that occupy much of the audience’s staring-at attention, though we frequently have little chance of ‘reading’ character therein. In a screenshot such as Figure 11, the face’s object status is therefore played up, not only in relation to Christiane-as-mannequin, but also because of the other faces in the shot, the photograph and painting, each of which bring their own potential significations. In this light, Biernoff’s discussion of Hans Bellmer’s dolls and of Franju’s direction as puppetry is enlightening for another reason: faces in this film are twisted and modified under the camera’s gaze as surely as under the scalpel. The fact that they are, to this extent, equivalent to other aspects of the *mise-en-scène* – exchangeable, modifiable, and free-floating as signifiers – is surely a linchpin of the film’s remarkably enduring shock affect. It is also the feature that permits the contested cultural histories of face transplantation Biernoff traces to find their place within this film, at once evoking medical, gothic, and satirical aspects of this gaze, drawn in large part from the influences she identifies, from surrealist film to the Grand Guignol, but also I would add from decades of facial play at work in horror cinema and other genres.

 Biernoff is certainly correct to position face transplantation within the history of the extreme makeover, and especially to associate the release of Franju’s film with the increasing prevalence of plastic surgery. But she is also correct to note this as just one part of a wide-ranging set of cultural anxieties and predispositions that potentially find focus within this film, something which I believe can only occur once some of our deep-engrained ‘certainties’ about the links between face and character have been effectively destabilised within the stylistic and facial economy of the film.

 Like Biernoff’s earlier work focussed on facial injury and surgery, I find the deep historicisation of this argument to be compelling and convincing, though, inevitably, in work of this kind that reaches simultaneously in so many different directions, more questions are posed than answered. If this article is a preface to a longer work, perhaps one with Fiedler’s long historical scope, I will most certainly look forward to reading it.

I have read this submission. I believe that I have an appropriate level of expertise to confirm that it is of an acceptable scientific standard.

## References

[1] ElsaesserTHagenerM: Film Studies: An Introduction through the Senses, 2nd edn. *London: Routledge*.2015;

[2] FiedlerL: Freaks: Myths and Legends of the Secret Self. *London: Simon and Schuster*.1979;

# Peer review: Referee response for version 1

**DOI:** 10.21956/wellcomeopenres.15846.r33110

**Published:** 2018-05-29

**Authors:** Fay Bound Alberti

**Affiliations:** 1 Queen Mary University of London, London, UK

Face transplants are an increasingly prevalent yet contentious form of modern transplantation surgery. Their meanings and practice are shrouded in complex ethical, practical and emotional concerns, yet their history is only recently being addressed. In this article, Suzannah Biernoff explores the neglected cultural context in which facial transplantation is imagined, figured and culturally represented. This is an important contribution to a growing field, in which I acknowledge a shared interest. In my own forthcoming book on face transplants, I deal specifically with their emotional and societal impact. I also have a great deal of respect for Biernoff’s pathbreaking work on the history of facial disfigurement, for which she is perhaps best known.  

 Biernoff’s article draws together, with characteristic elegance, this previous work on facial disfigurement and a considered, intellectual approach to the visual pre-history of the face transplant. She also explores the longue durée of cosmetic surgery, as Sander Gilman has done, and considers the recurring motif of the transplanted organ across times and cultures. There is, perhaps, something disjointed about moving from the 5 ^th^century BC to the present day; yes, it is the case that surgeons practising today couch their surgical inheritance within a long intellectual trajectory, and that the examples given have become canonical in this practice. Yet by reproducing these examples, Biernoff’s approach is similarly homogenous. Not all surgeons believe, as Peter Butler is cited to suggest, that plastic and reconstructive and cosmetic surgery are essentially one and the same; there are important philosophical, institutional and ideological distinctions that form part of a professional identity for many modern surgeons.

 In terms of the material studied, Biernoff is sensitive to nuance. She weaves together a cultural narrative about the pre-history of face transplants that includes the famous sixteenth-century repairer of noses Tagliacozzi as an instance of myth-making around facial reconstruction; this is important because most accounts of plastic and reconstructive surgery do start with Tagliacozzi, or with the facial flaps of Sushruta Samhita in 600BCE. There is a difference between noses and faces, however; damage to and amputation of noses has been a widely recognised form of corporeal punishment in the past, and the gendered, sexualised implications of this kind of deliberate mutation are lost if we consider all forms of facial reparation as the same. 

 When dealing with the ‘myth and legend’ around face transplant, Biernoff is careful to separate cultural mythologies – such as the ‘miracle of the black leg’ that is so often cited in transplantation stories – from the function of such accounts, which is to legitimize the ‘divine’ goal of transplantation. While we have the usual references to *Frankenstein*or *Face/Off*as part of the cultural backdrop of anxieties about surgeons playing God, Biernoff also provides a detailed exploration of the first cinematic portrayal of face transplantation, in Georges Franju’s classic *Les Yeux sans Visage*( *Eyes without a Face, *1959), and links the characters in that work to contemporary medical practitioners and biologists. It is in using these sources that Biernoff’s writing seems to come into its own with a sensitive and imaginative engagement with the practices of creative production. Rather imaginatively, Biernoff also brings animal vivisection and experimentation to the table, and this is original, as most accounts link face transplant surgery to cosmetic enhancement or transplantation, rather than to animalism and experimentation. 

 There are some references omitted, that I would have expected to be included, most notably Sharrona Pearl’s *Face/On: Face Transplants and the Ethics of the Other* ^1^. Pearl similarly situates the history of the face transplant within this broader cultural context of fears about transplantation and medical firsts; her book contains a chapter on ‘Losing Face in Film’ which also focuses on *Les Yeux sans Visage*, as well as other cultural reference points: *Seconds*(USA, 1966) and *The Face of Another*(Japan, 1966). Many other films are identified as key to facial identity during the same period (p.55) but these (forgive me) don’t make the cut. Pearl similarly addresses the significance of these films for the cultural role of the doctor and the medical advance, and she also tests out the extraordinary indexicality of the face as a reference point for humanity and identity, themes that are implicit, rather than explicit in Biernoff’s article. 

 I would have liked to have read more about the changing significance of the face in the lead up to the first transplant. This is promised in the abstract, yet not really addressed in the article. One of the most challenging aspects of face transplant surgery is that it takes place on the face, perhaps the most symbolically-laden part of the human body, as Pearl has shown. I was left with a lot of intriguing questions: what changes in cultural perceptions of the face took place between the time of Shushruta and Peter Butler? What are the disconnects and the differences that disturb the linear narrative being critiqued? And how does the Surrealist project, with its affinities with the stylistics of horror, create and engage with an audience for films like *Les Yeux sans Visage*? There seems to be a key story here, in this intersection between audience and creator, that is lost in the broad brushstrokes of this article. I suspect that is a story that Biernoff could tell better than anyone else.  

I have read this submission. I believe that I have an appropriate level of expertise to confirm that it is of an acceptable scientific standard.

## References

[1] Sharrona Pearl: Face/On: Face Transplants and the Ethics of the Other. *Chicago University Press*.2017;
